# Optimizing a high‐sensitivity NanoLuc‐based bioluminescence system for in vivo evaluation of antimicrobial treatment

**DOI:** 10.1002/mlf2.12091

**Published:** 2023-12-20

**Authors:** Weilong Shang, Zhen Hu, Mengyang Li, Yuting Wang, Yifan Rao, Li Tan, Juan Chen, Xiaonan Huang, Lu Liu, He Liu, Zuwen Guo, Huagang Peng, Yi Yang, Qiwen Hu, Shu Li, Xiaomei Hu, Jiao Zou, Xiancai Rao

**Affiliations:** ^1^ Department of Microbiology, College of Basic Medical Sciences, Key Laboratory of Microbial Engineering Under the Educational Committee in Chongqing Army Medical University (Third Military Medical University) Chongqing China; ^2^ Department of Microbiology, School of Medicine Chongqing University Chongqing China; ^3^ Department of Emergency Medicine, Xinqiao Hospital Army Medical University (Third Military Medical University) Chongqing China; ^4^ Department of Pharmacy, Xinqiao Hospital Army Medical University (Third Military Medical University) Chongqing China; ^5^ Department of Military Cognitive Psychology, School of Psychology Army Medical University (Third Military Medical University) Chongqing China

**Keywords:** antimicrobial evaluation, bioluminescence imaging, Nluc‐based luciferases, *Staphylococcus aureus*, therapeutic efficacy

## Abstract

Focal and systemic infections are serious threats to human health. Preclinical models enable the development of new drugs and therapeutic regimens. In vivo, animal bioluminescence (BL) imaging has been used with bacterial reporter strains to evaluate antimicrobial treatment effects. However, high‐sensitivity bioluminescent systems are required because of the limited tissue penetration and low brightness of the BL signals of existing approaches. Here, we report that NanoLuc (Nluc) showed better performance than LuxCDABE in bacteria. However, the retention rate of plasmid constructs in bacteria was low. To construct stable *Staphylococcus aureus* reporter strains, a partner protein enolase (Eno) was identified by screening of *S. aureus* strain USA300 for fusion expression of Nluc‐based luciferases, including Nluc, Teluc, and Antares2. Different substrates, such as hydrofurimazine (HFZ), furimazine (FUR), and diphenylterazine (DTZ), were used to optimize a stable reporter strain/substrate pair for BL imaging. *S. aureus* USA300/Eno‐Antares2/HFZ produced the highest number of photons of orange‐red light in vitro and enabled sensitive BL tracking of *S. aureus* in vivo, with sensitivities of approximately 10 CFU from mouse skin and 750 CFU from mouse kidneys. USA300/Eno‐Antares2/HFZ was a powerful combination based on the longitudinal evaluation of the therapeutic efficacy of antibiotics. The optimized *S. aureus* Eno‐Antares2/HFZ pair provides a technological advancement for the in vivo evaluation of antimicrobial treatment.

## Impact statement

Bioluminescence (BL) imaging with bacterial reporters has remained a powerful strategy to evaluate antibiotic treatment effects in vivo. In this study, we showed that NanoLuc (Nluc) worked effectively in bacteria, while the retention rate of plasmid in bacteria was low. A partner molecule enolase (Eno) was screened out from *Staphylococcus aureus* strain USA300 for stable fusion expression of Nluc‐based luciferases. We demonstrated that Nluc‐based luciferases showed superior sensitivity for BL imaging of *S. aureus* in vitro and in vivo. Our study may provide a model for generating other bacterial reporters for BL imaging applications.

## INTRODUCTION

Focal infections, such as skin abscesses, pyelonephritis, and pneumonia, as well as systemic infections, including bacteremia and sepsis, are commonly encountered in hospitals^1^, ^2^, ^3^, ^4^. The increase of bacterial antimicrobial resistance complicates the control of infectious diseases and results in high disability and mortality rates^5^. A study based on 471 million individual records and 7585 study‐location‐years revealed that six leading drug‐resistant bacteria, namely, *Escherichia coli*, *Staphylococcus aureus*, *Klebsiella pneumoniae*, *Streptococcus pneumoniae*, *Acinetobacter baumannii*, and *Pseudomonas aeruginosa*, were responsible for 3.57 million deaths in 2019^5^. If no action is implemented, then 10 million people will have died every year due to antimicrobial resistance by 2050^6^. The World Health Organization has suggested a priority list of antibiotic‐resistant bacteria for the discovery and development of new antibiotics^7^. Preclinical animal models are crucial in assessing the antimicrobial efficacy of new drugs and therapeutic regimens^8^, and a noninvasive and affordable strategy allowing for the accurate tracking of pathogens in vivo is urgently needed for drug screening.

Bioluminescence (BL) imaging relies on the catalysis of luciferase on its substrate, which is termed luciferin^9^, and has been commonly used for imaging target cells or reporting biological events in living animal models^10^, ^11^. More than 40 bioluminescent systems, including eukaryotic and prokaryotic luciferase‐based BL systems, have been established^12^. The classical and most applicable bacterial BL system is genetically encoded by the single *luxCDABE* operon derived from the insect pathogen *Photorhabdus luminescens* ^13^, ^14^, and such a system built into metabolically active bacteria can naturally emit blue‐green light with a maximum wavelength of 490 nm^13^, ^15^, ^16^. Previous studies showed that the *lux* system constructed in *S. aureus* can be used for drug discovery or the evaluation of antistaphylococcal efficacy in vitro, although the brightness of the *luxCDABE* reporter system is low^17^, ^18^. An improved *lux* operon, designated *ilux*, was generated in *E. coli* via coexpression of an additional flavin mononucleotide reductase to provide sufficient substrate and a mutant *lux* operon optimized by error‐prone mutagenesis of the wild‐type *luxCDABE* operon^19^. The brightness of the *ilux* system increases by approximately sevenfold compared with that of *lux* when expressed in *E. coli.* Moreover, the *ilux* system can achieve a single‐cell image in vitro. However, both *lux* and *ilux* emit a photon peak at 490 nm, and light wavelengths shorter than 600 nm decrease rapidly in mammalian tissue, thereby limiting the use of the *lux* system in vivo^9^, ^20^.

Many luciferases derived from diverse organisms, such as firefly luciferase (Fluc), which presents low catalytic activity with a peak emission of 578 nm^21^, *Renilla* luciferase (Rluc), which shows high catalytic activity but emits natively at wavelengths below 500 nm with a low bioluminescent quantum yield^22^, and luciferase (Luc) from the click beetle *Pyrophorus plagiophtalamus*, which produces red light with a long emission wavelength of >600 nm, have been selected to develop BL systems^23^. NanoLuc (Nluc) is a new small luciferase subunit (19 kDa) from the deep‐sea shrimp *Oplophorus gracilirostris* that demonstrates an ∼150‐fold increase in catalytic activity compared with other common luciferases, including Fluc and Rluc^24^. Nluc utilizes coelenterazine in an ATP‐independent reaction to produce blue light that peaks at 454 nm^24^. Nluc is rarely used in BL imaging because of its short‐wavelength light emission. Intramolecular BL resonance energy transfer (BRET) was applied, and a novel molecule, designated Antares, was designed by fusing Nluc with two domains of the cyan light‐excitable orange‐red fluorescent protein (CyOFP1) to produce photons with long wavelengths^22^, ^25^, ^26^. Antares is the most red‐shifted among the variants because it emits >430‐fold more red photons at >600 nm per molecule compared with Fluc^20^, ^21^, ^22^. A BRET‐based Antares2 reporter was developed by replacing the wild‐type Nluc with the high‐catalytic activity mutant Teluc (Nluc carrying D19S, D85N, and C164H mutations) in Antares to further improve BL signals in deep tissues^20^. The Antares2/diphenylterazine (DTZ) reporter can emit 3.8‐fold more photons above 600 nm than Antares/DTZ and 65‐fold stronger signals than Fluc/d‐luciferin while easily tracking tumor cells in deep tissues in vivo^20^. However, tracking bacterial cells with BL reporters in vivo is still a challenge.

Bioluminescent Nluc and LuxCDABE were expressed with plasmids in *S. aureus*, *E. coli*, and *P. aeruginosa* in the present study. Nluc presented higher BL signals than LuxCDABE in the tested bacteria. However, the retention rate of recombinant plasmids in bacteria was low. A carrier molecule enolase (Eno) was identified by screening of *S. aureus* strain USA300 for fusion expression of Nluc‐based luciferases, and three reporter strains, including USA300/Eno‐Nluc, USA300/Eno‐Teluc, and USA300/Eno‐Antares2, were generated. Further testing revealed that the USA300/Eno‐Antares2/hydrofurimazine (HFZ) combination enabled highly sensitive BL imaging for tracking *S. aureus* in vivo and was powerful in the longitudinal evaluation of the antimicrobial effect.

## RESULTS

### Nluc functioned differently in bacterial species and strains

The majority of luciferase reporters are constructed with plasmids^16^, ^23^, ^27^. The *nluc* gene was amplified from the plasmid pNL1.1 (Promega) and cloned into the *E. coli*–*S. aureus* shuttle plasmid pLI50^28^ under the control of the promoter of the *S. aureus fhuD2* gene that encodes a protein involved in iron metabolism^29^ to detect the performance of an engineered luciferase Nluc (19 kDa) from the deep‐sea shrimp *O. gracilirostris* in bacteria^24^. The bioluminescent *luxCDABE* operon of the bacterial insect pathogen *P. luminescens* from the plasmid pAKlux2.1 (Addgene) was also constructed into pLI50^16^, ^30^. The resulting plasmid, pFH‐*nluc* or pFH‐*lux* (Figure S1A,B and Table S1), was transformed in sequence into *E. coli* DH5α and *S. aureus* RN4220 and then transformed into *S. aureus* Newman and USA300 to achieve reporter bacteria. In addition, the *nluc* gene was directly fused with the gentamycin acetyltransferase gene in the pUCU24 plasmid^31^, and a *P. aeruginosa* reporter was also constructed by transforming pUCP24‐*nluc* into strain PAO1 (Figure S1C).

**Figure 1:**
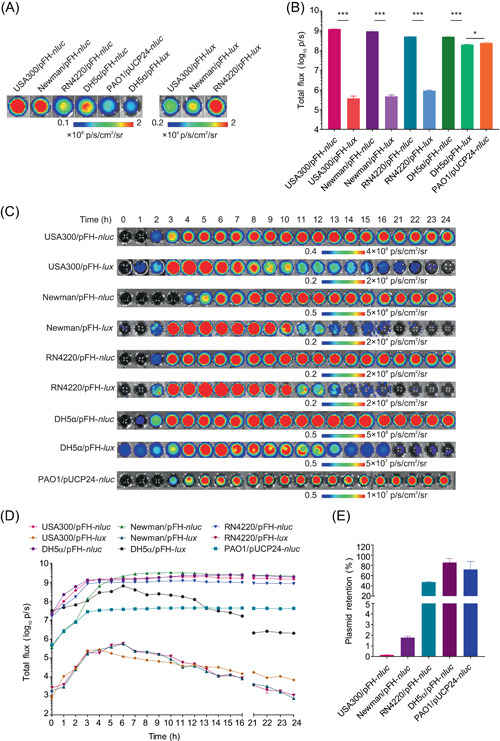
Comparison of Nluc and LuxCDABE engineered bacteria for in vitro bioluminescence (BL) imaging. (A) Representative BL images of 50 µl of pFH‐*nluc‐* or pUCP24‐*nluc‐* transformed reporter bacterial culture (1 × 10^7^ CFU/ml) mixed with 50 µl of HFZ (100 µM) and 100 µl of pFH‐*lux*‐transformed reporter bacterial culture (5 × 10^6^ CFU/ml). (B) Quantification of total flux produced from reporter bacteria after 6 h of culture. Data are presented as mean ± standard error of the mean (SEM). Statistical significance was analyzed using the unpaired two‐tailed *t*‐test. *p < 0.05 and ***p < 0.001. (C) Change of BL signals of the reporter bacteria cultured within 24 h. The cultures of diverse bacteria were harvested at the time indicated. BL signals were measured by mixing 50 µl of 1:100 diluted pFH‐*nluc‐* or pUCP24‐*nluc‐*transformed bacterial culture with 50 µl of HFZ (100 µM) in a black 96‐well plate. The signals from the wells carrying 100 µl of 1:200 diluted pFH‐*lux‐*transformed bacterial cultures were also determined using the IVIS® Lumina LT system. Representative BL images are shown. (D) BL intensity for each reporter strain over time. Data are presented as mean ± SEM. (E) Stability determination of plasmids in bacteria cultured in vitro. After 48 h of culture, the bacterial CFU was determined using the plate dilution method with a BHIA plate and BHIA supplemented with 10 µg/ml of Cm, or using an LB agar plate and an LB agar plate supplemented with 100 µg/ml of Amp or 20 µg/ml of Gm. Each experiment was repeated at least three times in triplicate. The mean number of viable bacteria grown on BHIA/LB plates was adjusted to 100%, while the relative numbers of viable bacteria grown on BHIA/LB plates supplemented with Cm, Amp, or Gm were calculated and are indicated. Data are presented as mean ± SEM. Amp, ampicillin; BL, bioluminescence; CFU, colony‐forming unit; Cm, chloramphenicol; Gm, gentamicin; p/s, photons/seconds.

Bacterial reporters with the *luxCDABE* operon construct can naturally produce blue‐green light, while a substrate is required for those with the *nluc* gene^14^, ^16^, ^32^. BL signals of the reporter bacteria with pFH‐*nluc* were determined by mixing 1 × 10^7^ CFU/ml of bacterial cells harvested at the mid‐logarithmic phase with an equal volume of substrate HFZ (100 µM). The staphylococcal *fhuD2* promoter‐controlled *nluc* gene functioned correctly in *S. aureus* strains RN4220, Newman, and USA300 (Figure 1A). Notably, we revealed that the pFH‐*nluc* construct also functioned correctly in *E. coli* (Figure 1A), thereby indicating the activity of the *fhuD2* gene promoter across bacterial species. The *P. aeruginosa* reporter carrying pUCP24‐*nluc* could also catalyze HFZ to produce brightness (Figure 1A). However, the brightness of reporter strains varied even under the same culture conditions. As shown in Figure 1B, *S. aureus* Newman/pFH‐*nluc* and USA300/pFH‐*nluc* generated similar brightness, which was approximately twofold higher than that of RN4220/pFH‐*nluc* and DH5α/pFH‐*nluc* and around fivefold higher than that of PAO1/pUCP24‐*nluc* after 6 h of culture. The bacterium‐carried *nluc* construct generated considerably higher brightness than those with *lux* constructs (Figure 1A,B). BL signals of reporters may be affected by the growth phase of bacteria. We revealed that pFH‐*nluc* or pUCP24‐*nluc* reporter strains produce relatively stable brightness compared with the strain carrying the pFH‐*lux* plasmid, which decreased substantially after 9 h of culture (Figure 1C,D). Moreover, Nluc‐catalyzed HFZ produced ~2–10^6^‐fold higher BL signals than its *lux* operon counterpart at different growth phases (Figure 1D).

A recombinant plasmid will be progressively lost in the absence of antibiotic‐selective pressure^27^. We then detected the stability of plasmids in the reporter strains grown in vitro using the plate dilution method^27^. Compared with the stability of pFH‐*nluc* in *E. coli* DH5α (86%) and pUCP24‐*nluc* in *P. aeruginosa* PAO1 (72%), only 0.15%–47.4% of *S. aureus* strains retained the pFH‐*nluc* plasmid after 48 h of culture (Figure 1E). In vivo studies also showed that only 0.0018% of USA300/pFH‐*nluc* exists in the kidneys of mice 5 days postinfection through the tail vein (Figure S1D). Overall, these data demonstrated that Nluc achieved better BL performance than LuxCDABE in both Gram‐positive and Gram‐negative bacteria. However, the brightness of reporters varied in different bacterial species and strains. The stability of the *nluc* construct also varied in bacterial strains and species, with the minimum stability of pFH‐*nluc* observed in *S. aureus* USA300. These results highlighted the high requirement for BL reporter system optimization.

### Screening of *S. aureus* partner molecules for fusion of Nluc‐based luciferases

**Figure 2:**
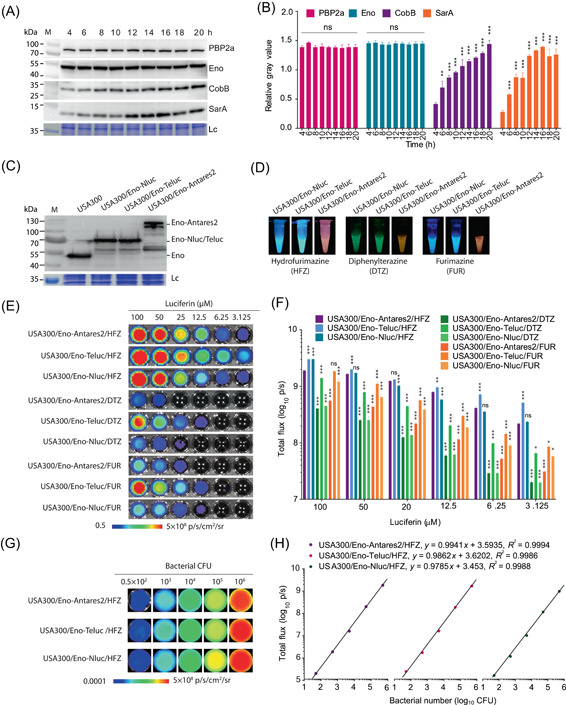
Construction of stable and functional Nluc‐based luciferase‐producing *Staphylococcus aureus* reporter strains. (A) Western blot analysis of the expression of PBP2a, Eno, CobB, and SarA in *S. aureus* USA300 over time. The protein gel served as a loading control (Lc), and the molecular weights of the protein markers (M) are indicated on the left. (B) Semi‐quantitative analysis of the gray values of the indicated bands in each lane of (A) using ImageJ software. Data are presented as mean ± SEM. Statistical significance was calculated by two‐way analysis of variance (ANOVA); ns indicates no significance, **p < 0.01, and ***p < 0.001. (C) Western blot analysis of Eno fusion proteins in the total cell lysates using mouse anti‐Eno polyclonal antibodies. (D) Bioluminescence (BL) photographs of different *S. aureus* reporter strain and substrate combinations. (E) Representative BL images of 50 µl of *S. aureus* reporter strains (1 × 10^7^ CFU/ml) mixed with 50 µl of different doses of HFZ, FUR, or DTZ (3.125–100 µM). (F) Quantification of total flux produced from *S. aureus* reporter strains with different concentrations of substrates. Data are presented as mean ± SEM. Statistical significance was analyzed by two‐way ANOVA; ns indicates no significance, *p < 0.05, **p < 0.01, and ***p < 0.001. (G) Images of BL signals from USA300/Eno‐Nluc, USA300/Eno‐Teluc, and USA300/Eno‐Antares2 over a range of bacterial loads with 50 µl of HFZ (100 µM) in the black 96‐well plates. (H) Good correlation between the BL intensity and bacterial number of *S. aureus* USA300/Eno‐Nluc, USA300/Eno‐Teluc, or USA300/Eno‐Antares2. The experiment was repeated at least three times in triplicate. Data are presented as mean ± SEM. *x*, bacterial number; *y*, BL intensity.

The plasmid‐mediated *nluc* (pFH‐*nluc*) in the hypervirulent methicillin‐resistant *S. aureus* strain USA300, which was isolated in 2000 and has become a major source of community‐acquired infections worldwide^33^, was unstable in vitro and in vivo (Figures 1E and S1D). We next attempted to construct a stable reporter strain by inserting the gene encoding Nluc‐based luciferase into the chromosome of USA300. A fusion carrier molecule is necessary for the stable expression of exogenous genes^34^. We hypothesized that a partner for fusion expression of Nluc‐based luciferases must meet several criteria: (i) expression is stable during the entire growth phase; (ii) expression is unaffected by stress conditions, such as antibiotic treatment; and (iii) fusion with the exogenous gene of interest fails to arrest bacterial growth. Among the proteins that met these criteria, four evolutionarily conserved proteins in *S. aureus*, namely, penicillin‐binding protein 2a (PBP2a)^35^, enolase (Eno)^36^, NAD‐dependent deacetylase (CobB)^37^, and the global regulator SarA^38^, were randomly selected to determine their availability as a fusion partner. Western blot analyses revealed that the expression of PBP2a and Eno was stable throughout the growth phase of *S. aureus* USA300, while that of CobB and SarA varied (Figure 2A,B). PBP2a, a target of β‐lactams, increased in expression in response to oxacillin treatment but demonstrated the opposite effect with Eno (Figure S2A). A study showed that Eno acts as a predominant component of *S. aureus* membrane vesicles and can carry foreign proteins to membrane vesicles^39^. This finding implied that Eno could be an appropriate candidate for the delivery of exogenous Nluc‐based luciferases.

Genes that encode Nluc‐based luciferases, namely, Nluc, Teluc, and Antares2, were chemically synthesized and genetically fused in‐frame with the 3ʹ‐terminal of *eno* in the chromosome of *S. aureus* USA300 to achieve USA300/Eno‐Nluc, USA300/Eno‐Teluc, and USA300/Eno‐Antares2 reporter strains, respectively (Figure S2B). The expected fusion proteins of Eno‐Nluc, Eno‐Teluc, and Eno‐Antares2 were confirmed via Western blot analysis (Figure 2C). The soluble substrate HFZ with acceptable solubility^11^, the imidazopyrazinone substrate furimazine (FUR)^24^, and the coelenterazine analog DTZ^9^ were used to detect the BL intensity of reporter strains in vitro. The results showed that *S. aureus* reporter strains USA300/Eno‐Nluc, USA300/Eno‐Teluc, and USA300/Eno‐Antares2 could catalyze HFZ, FUR, and DTZ to produce macroscopic light (Figure 2D). Furthermore, the hemolytic activities and growth curves of all reporter strains of interest were similar to those of the wild‐type strain USA300 (Figure S2C,D). We demonstrated that all three luciferase reporter strains could catalyze HFZ to generate stable BL signals within 24 h of testing (Figure S2E,F). The brightness of luciferase reporter/HFZ pairs was also assessed under different temperatures, pH, and other physiochemical conditions such as primary solubilizing agent PEG‐300^11^, cell culture medium RPMI‐1640^40^, RPMI‐1640 plus 10% fetal bovine serum, phosphate‐buffered saline, 2‐[4‐(2‐hydroxyethyl)‐1‐piperazinyl] ethanesulfonicacid (HEPES) buffer, Tris‐EDTA buffer, and normal saline (NS, 0.9% NaCl). The results demonstrated that all reporter combinations were stable at 30°C–40°C, pH 6–8 (Figure S2G–J), and produced higher BL signals under other physiochemical conditions (Figure S2K,L).

The bioluminescent intensities of strains USA300/Eno‐Nluc, USA300/Eno‐Teluc, and USA300/Eno‐Antares2 were evaluated with different concentrations of substrates, given their high stability. As shown in Figure 2E,F, reporter strains catalyzed HFZ to produce the highest brightness signals, followed by FUR and DTZ. Although the USA300/Eno–Teluc/HFZ pair generated the highest total emission, the USA300/Eno–Antares2/HFZ pair emitted >2.5‐fold more photons of orange‐red light than other pairs (Figures 2D and S3A–C), and HFZ did not inhibit the growth of USA300/Eno‐Antares2 (Figure S3D,E). We also found that the BL signals of USA300/Eno‐Nluc/HFZ, USA300/Eno‐Teluc/HFZ, and USA300/Eno‐Antares2/HFZ were highly correlated with the total viable bacterial CFU counts in vitro (*R* ^2^ > 0.99) (Figure 2G,H). However, even low doses of toxic compounds, including nitrogen mustard^41^, dimethyl sulfoxide, and the antibiotics erythromycin (ERM) and vancomycin (VAN), could inhibit the growth of *S. aureus* USA300/Eno‐Antares2 and result in BL signal reduction (Figure S3F–H). These results indicated that the *S. aureus* USA300/Eno‐Antares2/HFZ pair had potential application in vivo, and any factor that affects bacterial growth had an effect on the BL signal.

### USA300/Eno‐Antares2/HFZ enabled sensitive BL imaging for tracking *S. aureus* in a mouse bacteremia model

**Figure 3:**
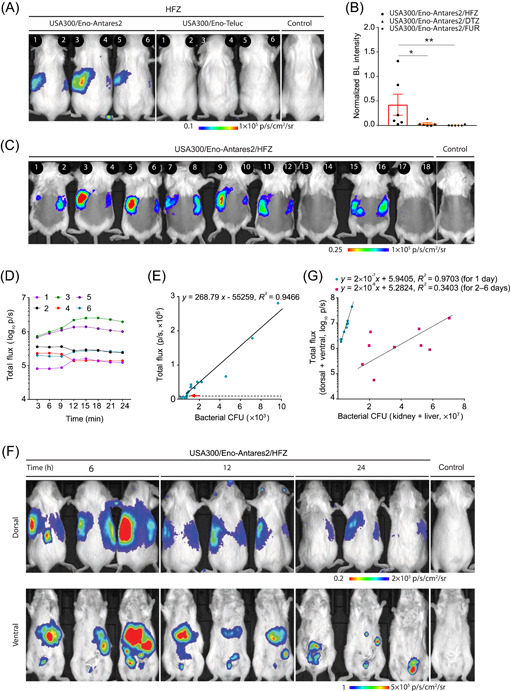
The USA300/Eno‐Antares2/HFZ pair is superior to other pair combinations for deep‐tissue bioluminescence (BL) imaging. (A) BL imaging of BALB/c mice that were infected intravenously with 1 × 10^7^ CFU of USA300/Eno‐Antares2 or USA300/Eno‐Teluc. After 24 h of infection, the mice (*n* = 3) were imaged after an intraperitoneal injection of 1 µmol of HFZ. 1 × 10^7^ CFU of USA300‐infected mice served as negative controls. Numbers 1, 3, and 5 represent the left kidneys, and 2, 4, and 6 indicate the right kidneys of the challenged mice. (B) Quantitative comparison of the normalized BL intensity of USA300/Eno‐Antares2 with diverse substrates indicated. Data are presented as mean ± SEM. Statistical significance was analyzed using the two‐tailed Mann–Whitney *U* test, *p < 0.05 and **p < 0.01. (C) Determination of BL sensitivity. BALB/c mice (*n* = 9) were each intravenously inoculated with 1 × 10^6^ CFU of USA300/Eno‐Antares2, and 1 µmol of HFZ was administered after 6 h of infection. In vivo BL signals were measured. *S. aureus* USA300‐infected mice served as negative controls. The left and right kidneys of mice are shown with specific numbers as indicated. (D) BL intensities of three representative mice to monitor signal decay over time (left and right kidneys, respectively). (E) Correlation between in vivo BL signals and bacterial loads in the right and left kidneys; the red arrow denotes the lowest CFU (750) detected by BL imaging. *x*, bacterial CFU; *y*, BL intensity. (F) BL images of the dorsal and ventral sides of mice (*n* = 3/group) at the time indicated. *S. aureus* USA300‐infected mice served as negative controls. (G) Correlation between total BL signals in vivo of the dorsal and ventral sides of mice and total bacterial CFU counts in kidneys and livers. The linear regression lines and correlation coefficients for the time span indicated are shown. *x*, bacterial CFU; *y*, BL intensity.

USA300/Eno‐Teluc/HFZ and USA300/Eno‐Antares2/HFZ pairs showed excellent performance in vitro, especially regarding total BL emission and orange‐red light photon production (Figures 2D–F and S3A–C). The engineered USA300/Eno‐Teluc and USA300/Eno‐Antares2 strains were chosen to infect BALB/c mice intravenously through a tail‐vein injection, and different substrates were individually administered to evaluate the performance of reporter strain/substrate pairs for *S. aureus* deep‐tissue BL imaging. BL signals could be detected in the kidneys on the dorsal sides of mice challenged with *S. aureus* USA300/Eno‐Antares2/HFZ, USA300/Eno‐Antares2/DTZ, USA300/Eno‐Antares2/FUR, and USA300/Eno‐Teluc/DTZ, but not in the kidneys of mice injected with USA300/Eno‐Teluc/HFZ and USA300/Eno‐Teluc/FUR (Figures 3A and S4A,B). However, bacterial loads in the kidneys of infected mice were comparable in each group (Figure S4C). The BL intensity was normalized with the total BL signal relative to the total viable bacterial count in each kidney. The results showed that the normalized BL intensity of the USA300/Eno‐Antares2/HFZ pair was the highest among all reporter/substrate pairs (Figures 3B and S4D).

The sensitivity of deep‐tissue BL imaging is usually determined using bacterial colonization from animal kidneys. However, the bacterial count from kidneys varies among individual animals despite being challenged with the same number of bacteria^23^. Mice were infected with 1 × 10^6^ CFU of USA300/Eno‐Antares2 via a tail‐veil injection, and in vivo BL signals were captured 6 h postinfection after administering 1 µmol of HFZ to detect the sensitivity of USA300/Eno‐Antares2/HFZ for deep‐tissue BL imaging. Compared with the BL signals of mice challenged with USA300 (negative control), the BL signals from the dorsal sides of mice were variable, and signals were absent in two of the infected mice (Figure 3C). The BL signals of three infected mice were collected every 3 min for 24 min after administering HFZ to monitor signal decay over time. The results demonstrated that BL signals were stable in vivo (Figure 3D). Bacterial loads in the right and left kidneys of mice 6 h postinfection were determined, and a satisfactory correlation between BL signals and viable bacterial numbers within 6 h of infection was observed (*R* ^2^ = 0.9466) (Figure 3E). A sensitivity of approximately 750 CFU from the kidneys was detected via in vivo BL imaging, and this value was 40‐fold higher than that of the *S. aureus luc* reporter system^23^.

Emitted in vivo BL signals are inaccurate during invasive and deep‐seated bacterial infections, and the actual in vivo bacterial load is underestimated^42^, ^43^. BL signals for deep‐tissue BL imaging may vary on the ventral and dorsal sides of experimental mice^23^. Therefore, BL signals were collected from kidneys on the dorsal side and livers on the ventral side of mice at 6, 12, and 24 h and 2, 4, and 6 days after being challenged with 1 × 10^7^ CFU of USA300/Eno‐Antares2 (Figures 3F and S4E). Bacterial burdens in the kidneys and livers were also determined, and the normalized BL intensity was calculated with the total BL signals (dorsal side plus ventral side) divided by the total number of viable bacteria (kidney plus liver). Notably, we revealed that in vivo BL signals of USA300/Eno‐Antares2 were highly correlated with the numbers of bacterial CFU within 24 h of infection (*R* ^2^ = 0.9703 for 1 day, Figure 3G), but not after more than 1 day (*R* ^2^ = 0.3403 for 2–6 days, Figure 3G). Ex vivo bacterial CFU counts from the kidneys and livers of infected mice dynamically changed over time (Figure S4F), and abscesses formed in the kidneys of mice 6 days after infection (Figure S4G). We speculated that the formation of an abscess may impair BL signals by impeding the entrance of Nluc substrate. Therefore, the reporter system of USA300/Eno‐Antares2/HFZ could provide a useful tool for monitoring deep‐seated bacterial dissemination during the early stages of bacteremia and mainly within 24 h of postinfection.

### BL imaging of USA300/Eno‐Antares2/HFZ in the mouse skin infection model

**Figure 4:**
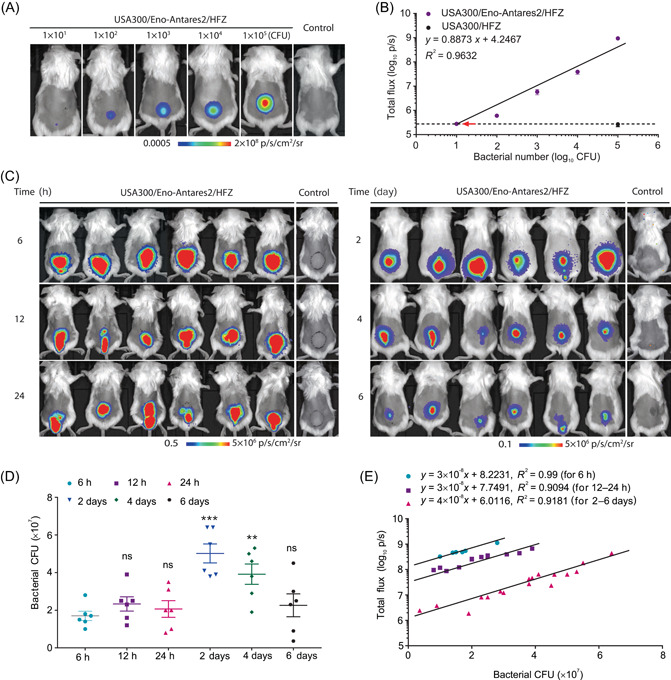
BL imaging of the USA300/Eno‐Antares2/HFZ pair in mouse skin infection. (A) Representative BL signals in mouse skins administered with 1 × 10^1^–1 × 10^5^ CFU of USA300/Eno‐Antares2, respectively. USA300 injected mice were served as negative controls. Each animal was anesthetized and then subcutaneously administered with 100 µl of mixture containing 50 µl of the indicated amount of the USA300/Eno‐Antares2 reporter strain and 50 µl of substrate HFZ (100 µM) (*n* = 3/group). (B) Quantitative analysis of BL signals produced 5 min after injection of reporter strain/HFZ. The red arrow indicates the lowest CFU capable of being detected in mouse skin by in vivo BL imaging (10 CFU). Data are presented as mean ± SEM. *x*, bacterial number; *y*, BL intensity. (C) Mice (*n* =  6/group) were subcutaneously infected with 1 × 10^7^ CFU of the reporter strain USA300/Eno‐Antares2, then 100 µl of HFZ (100 μM) was injected around the abscess, and BL signals at the indicated time after infection were captured. Mice administered 1 × 10^7^ CFU of the wild‐type USA300 and 100 µl of HFZ (100 μM) served as negative controls. (D) Bacterial CFU counts from the skin of mice at 6, 12, and 24 h, and 2, 4, and 6 days after infection with 1 × 10^7^ CFU of USA300/Eno‐Antares2. Data are presented as mean ± SEM. Statistical significance was analyzed by one‐way analysis of variance, ns indicates no significance, **p < 0.01, and ***p < 0.001. (E) Correlation between in vivo total BL signals and the viable bacterial loads in the skin of mice at 6 h, 12–24 h, and 2–6 days postinfection of USA300/Eno‐Antares2. The linear regression lines and correlation coefficient values are shown. *x*, Bacterial CFU; *y*, BL intensity.

*S. aureus* frequently causes skin and soft tissue disorders, such as impetigo, folliculitis, cellulitis, and wound infections^18^. We next assessed the BL intensity of the USA300/Eno‐Antares2/HFZ pair as a quantitative approach for evaluating the skin colonization of *S. aureus* based on the high‐brightness signals of the USA300/Eno‐Antares2/HFZ pair in vivo (Figures 3F and S4E). A mouse skin infection model was constructed by injecting the USA300/Eno‐Antares2 reporter strain into the skin on the dorsal side of the back^38^. Mice were injected subcutaneously with a mixture containing different CFU of bacteria (1 × 10^1^–1 × 10^5^ CFU) and an equal volume of HFZ (100 μM) to examine the in vivo sensitivity of BL imaging in the mouse skin. As shown in Figure 4A, 1 × 10^1^–1 × 10^5^ CFU of reporter strain‐challenged mice showed dose‐dependent focal BL signals. The total BL intensity (log_10_) was linearly correlated with the number of injected USA300/Eno‐Antares2 bacteria (*R* ^2^ = 0.9632) (Figure 4B). Therefore, sensitivity of approximately 10 CFU from the mouse skin was achieved through the optimized USA300/Eno‐Antares2/HFZ BL system. Three mice were subcutaneously challenged with USA300/Eno‐Antares2/HFZ and BL signals were collected every 5 min for 50 min to determine the optimal time for BL detection in skin. The results showed that in vivo BL signals from mouse skin were stable within 15 min and then decreased significantly (*p* < 0.01, Figure S5A).

Thirty‐six mice were injected subcutaneously with 1 × 10^7^ CFU of USA300/Eno‐Antares2 to explore the potential application of the USA300/Eno‐Antares2/HFZ system for detecting *S. aureus* in vivo. Body weights and skin abscess areas of infected mice were measured daily. The animals were imaged at 6, 12, and 24 h and 2, 4, and 6 days postinfection (*n* = 6 for each time point) after a subcutaneous injection of 100 µl of HFZ (100 µM) around the abscess. Infected mice were euthanized, and bacterial loads in the skin abscesses were determined after BL imaging. As shown in Figure S5B, skin abscess areas changed dynamically after being challenged with bacteria, while body weights were comparable among the groups (Figure S5C). BL signals could be detected in all infected mice at the time points tested. However, the BL intensity was variable with prolonged infection time (Figure 4C) and the ex vivo bacterial CFU counts changed (Figure 4D). Linear correlations between BL signals and USA300/Eno‐Antares2 bacterial loads were achieved at 6 h (*R* ^2^ = 0.99) and 12–24 h (*R* ^2^ = 0.9094), as well as 2–6 days (*R* ^2^ = 0.9181) postinfection (Figure 4E). The toxicity of HFZ to BALB/c mice was also investigated, and comparable body weights and serum interleukin 6 (IL‐6) and tumor necrosis factor‐α (TNF‐α) levels were observed (Figure S5D–F). This finding suggested that a strong association existed between in vivo BL signals and skin bacterial colonization. Hence, USA300/Eno‐Antares2/HFZ was a powerful reporter/substrate pair for tracking and quantifying bacteria in *S. aureus* skin infections.

### BL system of USA300/Eno‐Antares2/HFZ for the therapeutic efficacy evaluation of antibiotics against *S. aureus* skin infections

**Figure 5:**
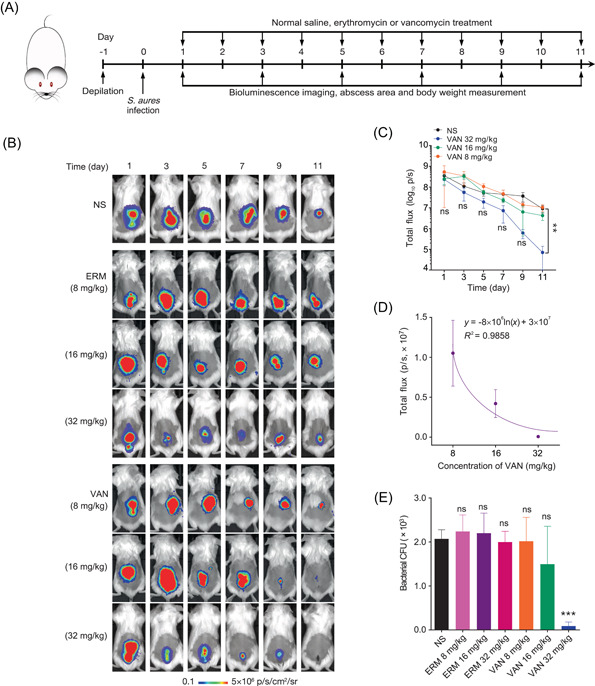
Evaluation of the treatment efficacy of antibiotics against *Staphylococcus aureus* skin infection using USA300/Eno‐Antares2 BL imaging. (A) Schematic diagram of the timeline in mouse skin model construction and antibiotic administration. The back hairs of BALB/c mice (*n* = 5/group) were depilated completely with sodium sulfide, and then 1 × 10^7^ CFU of USA300/Eno‐Antares2 were injected subcutaneously into each mouse. After 24 h of inoculation, the infected mice received vancomycin (VAN), erythromycin (ERM), or normal saline (NS) treatment for 11 days twice a day (every 12 h) through an intraperitoneal injection. During the therapeutic process, mice were imaged every other day, and the abscess area and body weight were also measured. (B) Longitudinal BL images of mice treated with NS and different concentrations of ERM or VAN as indicated. (C) BL intensities from each mouse in groups of NS‐treated and different concentrations of VAN‐treated were measured at the indicated time and presented. Data are presented as mean ± SEM. Statistical significance was analyzed using a two‐tailed Mann–Whitney *U* test; ns indicates no significance and **p < 0.01 relative to the NS‐treated group at the same time. (D) Correlation of BL intensities in the skin abscesses with different concentrations of VAN for treatment of 11 days postinfection of 1 × 10^7^ CFU of USA300/Eno‐Antares2. Data are presented as mean ± SEM. *x*, concentration of VAN; *y*, BL intensity. (E) Bacterial loads in the skins of mice after 11 days of VAN therapy. Data are presented as mean ± SEM. Statistical significance was analyzed by one‐way analysis of variance; ns indicates no significance and ***p < 0.001 relative to the NS‐treated group at 11 days postinfection.

Longitudinal and noninvasive monitoring of bacterial loads can provide key information about the disease pathogenesis and infectious course during antimicrobial therapy^8^, ^23^. We attempted to evaluate the efficacy of antibiotics against *S. aureus* skin infections with in vivo BL imaging on the basis of a high‐level correlation between in vivo BL signals and ex vivo CFU of the engineered USA300/Eno‐Antares2 reporter strain in the mouse skin infection model (Figure 4C–E). Wild‐type *S. aureus* USA300 is resistant to erythromycin (ERM, MIC > 128 µg/ml) but sensitive to vancomycin (VAN, MIC = 0.25 µg/ml)^33^, ^44^. Therefore, a mouse skin infection therapy model was constructed to investigate the capacity of the USA300/Eno‐Antares2/HFZ pair for the longitudinal evaluation of antibiotic efficacy against *S. aureus* in vivo (Figure 5A). As shown in Figure 5B, the administration of HFZ could track the location of the *S. aureus* USA300/Eno‐Antares2 reporter in mouse skin for 11 days. Consistent with the BL signals on the dorsal back of mice, the skin abscess areas in the groups treated with normal saline (NS) and different concentrations of ERM or VAN increased immediately after infection, and then gradually decreased over time (Figures 5B,C and S6A). BL intensities decreased faster in the 32 mg/kg of VAN‐treated group than in the NS‐treated group (Figure 5C). Further regression analysis demonstrated that BL intensities in the skin abscesses were linearly correlated with the concentration of VAN at 11 days (Figure 5D, *R* ^2^ = 0.9858), but not with ERM (Figure S6B). Compared with mice treated with NS, ERM, 8 mg/kg or 16 mg/kg of VAN, bacterial loads in the skin abscesses and IL‐6 levels in the sera were significantly lower in mice treated with 32 mg/kg of VAN at 11 days (Figures 5E and S6C). In addition, mouse body weights significantly increased in the 32 mg/kg VAN treatment cohort at 9 and 11 days compared with those in the NS treatment cohorts (Figure S6D). However, the comparable skin abscess sizes observed among the seven groups of infected mice (Figure S6E,F) indicated that the skin abscess size may be a poor index for bacterial skin colonization. Overall, these results demonstrated that VAN was an effective treatment, whereas ERM was not, and that *S. aureus* USA300/Eno‐Antares2 could be successfully used in the longitudinal evaluation of the therapeutic efficacy of antimicrobial agents in vivo. The optimized BL imaging system based on the USA300/Eno‐Antares2/HFZ pair provided a technological advancement for investigating the preclinical efficacy of anti‐*S. aureus* agents.

## DISCUSSION

The preclinical evaluation of antimicrobial efficacy in animal models is crucial for the development of new agents and therapeutic regimens^8^. In vivo BL imaging with bacterial reporters has remained a powerful strategy for the noninvasive and nondestructive monitoring of bacterial loads in the infection process^15^. In vivo imaging of bacterial pathogens using luciferases is challenging^23^. The high catalytic activity of Nluc enables the production of bright and sustained BL signals that peak at 454 nm^20^, ^24^. Several studies have attempted to engineer novel Nluc‐based luciferases with enhanced brightness and red‐light emission or develop substrates with satisfactory solubility for in vivo mammal cell imaging^11^, ^20^, ^32^. In the present study, the deep‐sea shrimp‐derived *nluc* gene and insect bacterial pathogen‐carried *luxCDABE* operon were constructed into plasmids, and their BL capacities were compared using diverse bacteria, including *S. aureus*, *E. coli*, and *P. aeruginosa*. We demonstrated that the BL brightness of Nluc and Lux varied according to the bacterial species and strains. The BL intensity of Nluc‐expressing bacteria was generally ~2–10^6^‐fold higher than that of Lux‐expressing bacteria (Figure 1D). However, the stability of recombinant plasmids was dependent on the bacterial species. Approximately 86% of *E. coli* DH5α and 72% of *P. aeruginosa* PAO1 could maintain pFH‐*nluc* and pUCP24‐*nluc* plasmids, respectively, while only 0.15%–47.4% of *S. aureus* strains could retain the pFH‐*nluc* plasmid after 48 h of culture in vitro (Figure 1E). Moreover, the retention rate of pFH‐*nluc* in *S. aureus* USA300 in vivo was low, and only 0.0018% of bacteria existed with plasmids 5 days postinfection of USA300/pFH‐*nluc* into mice through the tail vein (Figure S1D). Similar findings were also reported in the study of Bacconi et al., in which the stability of plasmids in *S. aureus* was improved by cloning toxin/antitoxin and partition systems into a plasmid of interest^27^. However, the complicated strategy involving stability modification of a certain plasmid has rarely been used.

Insertion of the target gene into the bacterial chromosome is an effective way to improve gene stability^45^. A suitable carrier molecule encoded by genomic DNA may be important for the successful expression of the exogenous gene in this case^34^. Therefore, an evolutionarily conserved Eno that plays an important role in *S. aureus* glycolysis and energy production^36^ was screened for the construction of Nluc‐based luciferases, including Nluc, Teluc, and Antares2, in *S. aureus*. The growth rate and hemolytic activity of *S. aureus* USA300 remained unaffected by the fusion of *nluc*, *teluc*, and *Antares2* with *eno* (Figure S2B–D). Furthermore, the fusion‐expressed Eno‐Nluc, Eno‐Teluc, and Eno‐Antares2 in *S. aureus* USA300 could catalyze various substrates to produce macroscopic light (Figure 2D). This finding indicated a successful approach for fusion expression of Nluc‐based luciferases with Eno in *S. aureus*. The carrier molecule‐based optimizing strategy may provide an acceptable example in the construction of other bacterial reporters, such as *E. coli* and *S. pneumonia* ^15^, ^19^, ^45^.

Photons emitted by luciferases shorter than 600 nm may strongly interact with mammalian tissues and show poor BL signals, particularly in deep tissues, during in vivo bacterial BL imaging^20^. Moreover, the use of Nluc‐based luciferases in vivo is limited by the poor water solubility and bioavailability of the substrate FUR or DTZ^11^, ^32^. The discovery of HFZ with excellent aqueous solubility allows for high‐dose substrate usage and improves light production in tracking the location and growth of tumor cells in mice^11^. We demonstrated that the USA300/Eno‐Teluc/HFZ pair generated the highest total BL emission, while the USA300/Eno‐Antares2/HFZ pair produced the most photons of orange‐red light in vitro when screening combinations of reporter strains and substrates (Figure S3C). This finding implied that the optimized and engineered bacterial reporters had high application potential. We then showed that the combination of *S. aureus* USA300/Eno‐Antares2 and HFZ was superior to other reporter/substrate pairs in BL imaging of deep tissues, such as the kidney and liver, using the mouse bacteremia model (Figure 3A,B). Approximately 750 CFU of USA300/Eno‐Antares2 in mouse kidneys could produce detectable BL signals (Figure 3E), thereby indicating a sensitivity 40‐fold greater than that of the *S. aureus luc* reporter system (about 3 × 10^4^ CFU from kidneys)^23^. Miller et al. showed a high‐level correlation between the in vivo BL data and ex vivo CFU counts of *S. aureus luc* reporter strain AH4775 from the kidneys of infected mice 16 h postinoculation (*R* ^2^ = 0.9924)^23^. Deep‐seated BL imaging revealed that signals from the ventral and dorsal sides of mice are variable^23^. Therefore, the normalized BL intensity was measured and calculated with the total BL flux (dorsal side plus ventral side) divided by the total number of bacterial CFU (kidney plus liver). Notably, we revealed that the total in vivo BL signals of *S. aureus* USA300/Eno‐Antares2 highly correlated with the ex vivo bacterial CFU within 24 h of infection (*R* ^2^ = 0.9703, curve fitting with the data for 6, 12, and 24 h after inoculation) but not at the time of more than 1 day (*R* ^2^ = 0.3403 for 2–6 days postinfection, Figure 3G). This phenomenon was likely attributed to the dynamic variation and distribution of bacteria in the internal organs of model animals (Figure S4F), individual differences in the host immune response, and substrate permeability inhibition by bacterial abscess formation (Figure S4G)^46^. Therefore, the optimal USA300/Eno‐Antares2/HFZ system may be useful to explore deep‐seated bacterial dissemination in the early stages, particularly within 24 h postinfection. New in vivo BL reporters with photon emission longer than 600 nm or novel substrates with high tissue or bacterial abscess permeability require further investigation to monitor deep‐seated bacterial dissemination.

Compared with the bacteremia model, the bacterial count from the mouse skin infection model confirmed that an acceptable correlation existed between BL data and bacterial CFU in the skin of mice at 6 h, 12–24 h, and 2–6 days postinoculation (Figure 4E). This result was consistent with those of previous studies^18^, ^23^. Additionally, the combination of USA300/Eno‐Antares2/HFZ showed high sensitivity of 10 CFU in monitoring *S. aureus* in mouse skin infections (Figure 4B). Therefore, the optimal USA300/Eno‐Antares2/HFZ pair was used for BL imaging to evaluate the antimicrobial efficacy of certain antibiotics against bacterial skin infections. The therapeutic model was designed on the basis of the resistant nature of *S. aureus* USA300, which is resistant to ERM and sensitive to VAN^33^, ^44^. The measured BL signals from infected skin samples remarkably decreased in mice treated with VAN compared with those treated with ERM or NS (Figures 5B,C, and S6A). BL intensities in the skin abscesses were linearly related to the VAN concentrations at 11 days (Figure 5D, *R* ^2^ = 0.9858). Correspondingly, the bacterial loads in the skin abscesses of 32 mg/kg VAN‐treated mice were substantially reduced compared with those of 8 mg/kg or 16 mg/kg of VAN‐, ERM‐, and NS‐treated mice at 11 days (Figure 5E). These findings suggested that VAN can be an effective treatment, while ERM is not. Different dosages of antibiotic treatment were used to confirm that the USA300/Eno‐Antares2/HFZ system could be successfully applied to evaluate the treatment performance of antimicrobial agents in vivo based on its high sensitivity (Figure 5A,B).

Overall, data from the present study demonstrated that Nluc‐based luciferases could catalyze their substrates to produce higher brightness signals than the classical *luxCDABE* system in BL imaging. The optimized USA300/Eno‐Antares2/HFZ pair improved the stability and brightness of BL signals in vitro and in vivo. Moreover, this combination provided reliable BL signals for monitoring the bacterial burden and lesion healing during antimicrobial treatment in a preclinical *S. aureus* skin infection model. The USA300/Eno‐Antares2/HFZ combination may be used as an in vivo BL evaluation tool for *S. aureus* infection at the early stages of systemic bacterial dissemination. The carrier molecule‐based optimization strategy for BL systems may provide a model for generating other bacterial reporters to establish noninvasive, stable, cost‐effective, and accurate animal models for focal and systemic antimicrobial treatment evaluation.

## MATERIALS AND METHODS

### Bacterial strains

Bacterial strains used in the present study are listed in Table S1. *E. coli* DH5α strain was purchased from TIANGEN Biotech (China). *S. aureus* USA300 strain FPR3757 (GenBank accession no. CP000255.1) was kindly provided by Min Li (Renji Hospital, Shanghai Jiao Tong University School of Medicine, Shanghai, China), Newman (NCTC 8178) was provided by Prof. Yu Lu (Jilin University, China), and RN4220 (NCTC 8325–4) was provided by Baolin Sun (University of Science and Technology of China). *P*. *aeruginosa* strain PAO1 was maintained in our lab. *E. coli* and *P*. *aeruginosa* strains were cultivated in Luria broth (LB) medium (Oxoid). *S. aureus* strains were cultured with brain–heart infusion (BHI) medium (Oxoid) or BHI agar (BHIA). When appropriate, the broth media or agar plates were supplemented with ampicillin (Amp, 100 μg/ml), gentamicin (Gm, 20 μg*/*ml), or chloramphenicol (Cm, 10 μg/ml).

### Construction of Nluc‐ and LuxCDABE‐producing bacterial strains

Plasmids used in the present study are listed in Table S1. A fragment of *fhuD2* gene promoter DNA (213 bp) was amplified from the genomic DNA template of *S. aureus* USA300 with the primer pair fhud2P‐F/R^27^, and inserted into the *E. coli*–*S. aureus* shuttle vector pLI50 to construct the pFH plasmid. Then, the DNA of *nluc* from the plasmid of pNL1.1 (Promega Corp.) or the *luxABCDE* operon from the pAKlux2.1 plasmid (Addgene) was amplified with the primer pair nluc‐F/R or lux‐F/R, and subsequently inserted into the pFH plasmid under the control of the *fhuD2* gene promoter to obtain pFH‐*nluc* and pFH‐*lux* plasmids, respectively. Similarly, the *nluc* gene was also amplified with primers nluc‐F1/R1 and fused with the gentamycin acetyltransferase gene of the pUCP24 vector^31^ to achieve the pUCP24‐*nluc* plasmid. The pFH‐*nluc* and pFH‐*lux* plasmids in *E. coli* DH5α were prepared, respectively, and transformed into *S. aureus* strains RN4220, Newman, and USA300. The pUCP24‐*nluc* plasmid was transformed into *P*. *aeruginosa* PAO1 to obtain Nluc‐producing strain PAO1/pUCP24‐*nluc*. All primers used in this study are listed in Table S2.

### In vitro BL imaging of Nluc‐ and LuxCDABE‐expressing bacterial strains

The imidazopyrazinone substrate FUR^24^, coelenterazine analog DTZ^9^, and soluble substrate HFZ^11^ were separately dissolved in a polyethylene glycol‐300 (PEG‐300) formulation made up of 10% glycerol, 10% ethanol, 10% hydroxypropylcyclodextrin, and 35% PEG‐300 in distilled water^11^. BL imaging was performed using the IVIS® Lumina LT Series III system (Perkin Elmer). Images were analyzed using Living Image 4.4 software. For BL imaging of Nluc‐producing bacteria in vitro, 50 µl of pFH‐*nluc* or pUCP24‐*nluc* transformed bacterial culture (1 × 10^7^ CFU/ml) was, respectively, mixed with 50 µl of diverse substrates (FUR, DTZ, and HFZ, 100 µM for each) in a black 96‐well plate and then measured. For BL imaging of LuxCDABE‐expressing bacteria, 100 µl of pFH‐*lux* transformed bacterial culture (0.5 × 10^7^ CFU/ml) was added to the wells of a black 96‐well plate and then detected by the IVIS® Lumina LT system. For the determination of BL signal stability, bacterial strains of interest were cultured in BHI or LB media at 37°C with shaking. The next day, 10 µl of each overnight bacterial culture was added to 2 ml of fresh BHI or LB broth and continued to culture at 37°C with shaking. Then, 10 µl of pFH‐*nluc* or pUCP24‐*nluc* plasmid‐carried bacterial culture was taken every hour up to 24 h, and separately added to 1 ml of Phosphate‐buffered saline (PBS) (1:100 dilution). After that, 50 µl of each sample was mixed with 50 µl of HFZ (100 µM) in a black 96‐well plate, and BL signals were measured with the IVIS® Lumina LT system. As for pFH‐*lux* transformed bacteria, a 10 µl sample taken from fresh culture at each hour was 1:200 diluted in PBS, and 100 µl of bacterial sample was added to the black 96‐well plate and determined. The wild‐type bacteria, including *E. coli* DH5α, *S. aureus* USA300, and *P. aeruginosa* PAO1, were used as negative controls. BL data were collected using the IVIS® Lumina LT system 1 min after the addition of a substrate. The parameters set for image acquisition were as follows: emission filter open for total BL; exposure time, 1–10 s; binning factor, 4; field of view, 12.5 cm; and f‐stop, 1.

### Plasmid stability in bacteria

The stability of plasmids in diverse reporter strains was detected using the plate dilution method as described^27^. Briefly, 10 µl of the overnight culture of pFH‐*nluc* transformed *S. aureus* USA300 was inoculated into 2 ml of fresh BHI broth in the absence of antibiotics and cultured at 37°C for 24 h, and this process was repeated once the next day. After 48 h of culture in BHI without antibiotics, 100 µl of the sample was 10‐fold serially diluted with fresh BHI, seeded onto a BHIA plate with or without antibiotics (Cm, 10 µg/ml), and incubated at 37°C for 18 h. Each experiment was repeated at least three times in triplicate. The mean number of viable bacteria grown on BHIA plates without antibiotics was adjusted to 100%, while the relative number of viable bacteria grown on BHIA containing Cm was calculated. The stability of plasmids in other reporter strains grown in vitro was also detected as described above.

To evaluate plasmid stability in vivo, BALB/c mice were intravenously injected with 1 × 10^7^ CFU of pFH‐*nluc* transformed *S. aureus* USA300 (approximately 30% lethal dose). After 5 days of infection, the infected mice were killed and the kidneys were homogenized in 1 ml of PBST (PBS with 0.1% TritonX‐100) using the instrument of Minibeadbeater 16 (Biospec)^47^. The homogenized sample was then serially diluted in PBS and seeded onto BHIA plates with or without Cm (10 µg/ml). The retention rate of the pFH‐*nluc* plasmid in *S. aureus* USA300 in vivo was calculated as described above.

### Screen of carrier molecules for expression of Nluc‐based luciferases in *S. aureus*

To construct stable Nluc‐based luciferase expressing *S. aureus* reporters, several evolutionarily conserved proteins in *S. aureus* USA300, such as PBP2a^35^, Eno^36^, CobB^37^, and the global regulator SarA^38^, were randomly selected to detect their potential as carrier molecules for fusion expression of exogenous luciferases. The expression levels of the candidate proteins in diverse growth phases of *S. aureus* USA300 were examined by Western blot. Briefly, the overnight culture of *S. aureus* USA300 was 1:100 diluted with 200 ml of BHI broth and cultivated at 37°C with shaking. Bacterial cells in 3 ml of culture harvested at 4, 6, 8, 10, 12, 14, 16, 18, and 20 h postinoculation were collected by centrifugation at 10,000*g* for 10 min at 4°C. The cell pellet was washed twice with PBS, resuspended in 1 ml of cold PBS supplemented with 1% (m/v) β‐mercaptoethanol (Sigma) and 1 mM PMSF (Beyotime). Cells were broken by addition of 0.1 mm diameter zirconia/silica beads and shaking on the Minibeadbeater 16 instrument (Biospec). Cell debris was removed after centrifugation at 10,000*g* for 10 min at 4°C. The protein concentration was determined using the Bradford Protein Assay Kit (Beyotime). The proteins of interest in *S. aureus* USA300 were separated by 12% sodium dodecyl sulfate polyacrylamide gel electrophoresis (SDS‐PAGE) and transferred onto a polyvinylidene difluoride (PVDF) membrane (GE Healthcare). The first mouse anti‐PBP2a, anti‐Eno, anti‐CobB, and anti‐SarA polyclonal antibodies and a secondary sheep anti‐mouse antibody coupled with horseradish peroxidase (Abmart) were used in the Western blot. The target protein signals were visualized by the SuperSignal West Atto Substrate (Thermo Fisher Scientific) and photographed.

### Construction of Eno‐fused Nluc‐based luciferase expressing *S. aureus* reporters

The nucleotide sequences of *nluc, teluc*, and *antares2* according to the codon usage bias of *S. aureus* USA300 were chemically synthesized by BGI‐Shenzhen (China) and cloned into the pUC18 plasmid to achieve pUC‐*nluc*, pUC‐*teluc*, and pUC‐*antares2*, respectively (Table S1). Based on the stable expression of Eno in *S. aureus* USA300, the *nluc, teluc*, and *antares2* genes in the plasmids of interest were individually amplified with polymerase chain reaction (PCR) and in‐frame fused with the *eno* gene within the genome of *S. aureus* USA300 using homologous recombinant strategy^38^. Briefly, the *nluc* gene was amplified using primers eno‐nluc‐F1/R1 from the pUC–*nluc* plasmid template. For site‐specific homologous recombination, the left and right homologous arms across the stop codon of the *eno* gene were designed and amplified from *S. aureus* USA300 genomic DNA with primers up‐eno‐F1/R1 and down‐eno‐F1/R1 (Table S2). The fusion fragment (left arm–*nluc*–right arm) was obtained by over‐lap PCR with the primer pair of up‐eno‐F1/down‐eno‐R1 and cloned into the temperature‐sensitive shuttle vector pBT2 with one step cloning strategy to generate pBT2‐*nluc* (Table S1). After transformation of the pBT2‐*nluc* plasmid into *S. aureus* USA300, the integration of plasmid into bacterial chromosome was induced by cultivating plasmid‐carried USA300 at 42°C for 20 h, followed by cultivating at 25°C for 20 h to achieve plasmid missing strains. The USA300/Eno‐Nluc strain was confirmed by PCR and DNA sequencing. Similar strategies were used to construct USA300/Eno‐Teluc and USA300/Eno‐Antares2, respectively. The expressions of the fusion proteins, such as Eno‐Nluc, Eno‐Teluc, and Eno‐Antares2, in the engineered *S. aureus* reporters were verified by Western blot.

### Hemolytic activity assay

To detect whether the fusion expression of Nluc‐based luciferase with Eno in *S. aureus* affects the bacterial pathogenicity, the hemolytic activity assay was performed. *S. aureus* reporter strains were each cultivated at 37°C to an optical density at 600 nm (OD_600_) of 2.0, the culture was 1:100 diluted in PBS, and 5 μl of bacterial suspension was spotted onto 5% (vol/vol) sheep blood agar plates and incubated at 37°C for 24 h. After culture, the plates were photographed.

### Bacterial growth curve

The growth curves of *S. aureus* engineering strains USA300/Eno‐Nluc, USA300/Eno‐Teluc, and USA300/Eno‐Antares2 were determined as previously described^48^. Briefly, *S. aureus* strains of interest were cultured in BHI broth at 37°C overnight with shaking, and 0.2 ml of each culture was added to 20 ml of fresh BHI broth in a sterile 50 ml flask. The OD_600_ values were measured every hour for 24 h after inoculation. The OD_600_ values over the cultivation time were used to draw the growth curves.

### Detection of BL signals of *S. aureus* reporter strains in tubes

*S. aureus* reporter strains USA300/Eno‐Nluc, USA300/Eno‐Teluc, and USA300/Eno‐Antares2 were separately cultured in BHI broth at 37°C to an OD_600_ of 2.0. Next, the bacterial cells were harvested by centrifugation at 10,000*g* for 10 min at 4°C and adjusted to 1 × 10^9^ CFU/ml with PBS. BL images of the test tubes containing 100 µl of *S. aureus* reporter suspension and 10 µl of individual substrate (HFZ, DTZ, or FUR, 3 mM for each) were taken in a dark room using a V40 digital camera (HONOR) with parameters of exposure time of 1 s, ISO 16,200, and f‐number of 1.9.

### Evaluation of the stability of *S. aureus* reporter strains in vitro

The strains of interest were adjusted to 1 × 10^6^ CFU/ml with PBS (pH 7.3), 50 µl of bacterial solution was mixed with 50 µl of HFZ (100 µM) in a thermostatic metal bath at different temperatures (0°C–50°C) or bacteria were prepared with PBS under different pH (4–10). BL signals were detected using the IVIS® Lumina LT system 1 min after the addition of a substrate.

The stability of *S. aureus* reporter strains was also tested in other physiochemical conditions, including PEG‐300 formulation, HEPES (1 M, pH 7.3), TE (1×, pH 7.3), NS, RPMI‐1640, or RPMI‐1640 plus 10% FBS.

### BL imaging of the engineered *S. aureus* reporter strains in vitro

To compare the BL intensity of the engineered *S. aureus* reporter strains in vitro, the strains of interest were cultured in BHI and adjusted to 1 × 10^7^ CFU/ml with PBS. A total of 50 µl of *S. aureus* suspension was individually mixed with an equal volume of the substrate at diverse concentrations (3.125, 6.25, 12.5, 25, 50, and 100 µM) in a black 96‐well plate and then BL signals were measured using the IVIS® Lumina LT system. To analyze the linear correlation between BL signals and bacterial numbers, *S. aureus* reporter strains USA300/Eno‐Nluc, USA300/Eno‐Teluc, and USA300/Eno‐Antares2 were cultured, and bacterial solutions of 1 × 10^2^ to 1 × 10^6^ CFU/ml in PBS were prepared, 50 µl of bacterial solution was mixed with 50 µl of HFZ (100 µM) in a black 96‐well plate; BL images and signals were collected using the IVIS® Lumina LT system 1 min after the addition of a substrate. Wild‐type *S. aureus* USA300 was used as a negative control. The parameters for image acquisition were as follows: emission filter open for total BL flux or a 591 ± 10 nm emission filter used for red BL signals; exposure time, 1 to 10 s; binning factor, 4; field of view, 12.5 cm; and f‐stop, 1.

To determine whether HFZ has any effects on the reporter strain, 0.2 ml of USA300/Eno‐Antares2 culture was added to 20 ml of fresh BHI broth containing diverse concentrations of HFZ (12.5, 25, 50, and 100 µM) in a sterile 50 ml flask. The OD_600_ values were measured every hour for 24 h after inoculation. The growth curve was drawn. Then, the bacterial number was counted using the plate dilution assay.

The toxicities of different concentrations of NM (1.25, 2.5, 5.0, and 10 µM), dimethyl sulfoxide (1.25% (v/v), 2.5%, 5%, and 10%), ERM (4, 8, 16, and 32 µg/ml), or VAN (0.125, 0.25, 0.5, and 1 µg/ml) to the USA300/Eno‐Antares2 reporter strain were also evaluated with black 96‐well plates in vitro.

### Emission spectra analysis of BL signals from the engineered *S. aureus* reporters

To determine the emission spectra of Eno‐fused Nluc‐based luciferases in *S. aureus*, the reporter strains of interest were separately cultured at 37°C to an OD_600_ of 2.0, and then bacterial cells were harvested by centrifugation at 10,000*g* for 10 min at 4°C and adjusted to 1 × 10^9^ CFU/ml with PBS. 100 µl of *S. aureus* suspension of USA300/Eno‐Nluc, USA300/Eno‐Teluc, or USA300/Eno‐Antares2 was mixed with 10 µl of individual substrate (FUR, DTZ, or HFZ, 3 mM for each) in a black 96‐well plate. After 1 min of reaction, the BL spectra were collected using a SpectraMax M2 (Molecular Devices) with 1‐nm increment from 400 to 700 nm. The spectra of Eno‐Teluc and Eno‐Antares2 were normalized to the Nluc emission peaked at 460 nm. The experiment was repeated three times in triplicate.

### Selection of the reporter strain/substrate pair for deep‐tissue BL imaging

Based on the emission spectra analysis in vitro, a mouse *S. aureus* bacteremia model was generated and used to screen the reporter strain/substrate combination for deep‐tissue BL imaging. Female BALB/c mice (*n* = 3 for each combination) were administered intravenously with 1 × 10^7^ CFU of the USA300/Eno‐Teluc or USA300/Eno‐Antares2 reporter strain. After 24 h, the infected mice were injected with 1 µmol of substrate (HFZ, DTZ, or FUR) into the peritoneal cavity. Wild‐type USA300‐challenged mice served as negative controls. BL signals in the kidneys from the dorsal sides of mice were detected using the IVIS® Lumina LT system 3 min after injection of a substrate^22^. Images were acquired every 3 min and the maximal signal data were chosen for subsequent analysis. The bacterial loads in the kidneys of infected mice were also determined using the plate dilution assay as described previously^45^. The normalized BL intensity was calculated by dividing the BL signals by the total number of viable bacteria in each kidney.

### BL imaging of *S. aureus* USA300/Eno‐Antares2 in a mouse bacteremia model

To determine the sensitivity of BL imaging of *S. aureus* USA300/Eno‐Antares2 systemic dissemination, BALB/c mice (*n* = 9) were infected intravenously with 1 × 10^6^ CFU of the reporter strain. After 6 h of infection, 1 µmol of HFZ was intraperitoneally injected, and in vivo BL signals were obtained from the dorsal sides of mice 3 min after injection of the substrate. To monitor signal decay over time, the BL signals on the dorsal sides of mice were measured every 3 min up to 24 min. The bacterial loads in the kidneys of infected mice were determined using the plate dilution assay. In vivo deep‐tissue BL sensitivity was determined as the lowest bacterial CFU in the kidneys of mice that can still emit a bioluminescent signal.

To evaluate the correlation between deep‐tissue BL signals and bacterial CFU numbers, BALB/c mice (*n* = 3/group) were infected intravenously with 1 × 10^7^ CFU of *S. aureus* USA300/Eno‐Antares2. Then, mice were intraperitoneally administered with 1 µmol of HFZ at 6, 12, and 24 h, and 2, 4, and 6 days postinfection. The in vivo BL signals were collected from the dorsal and ventral sides of mice. The bacterial loads in the kidneys and livers of infected mice were also determined using the plate dilution assay^47^. The total flux from in vivo deep tissues (dorsal + ventral) and total bacterial CFU (kidney + liver) were used to assess the correlation between deep‐tissue BL signals and bacterial loads in mice.

### BL imaging of *S. aureus* USA300/Eno‐Antares2 in a mouse skin infection model

To investigate the lower limit of sensitivity for BL imaging of *S. aureus* USA300/Eno‐Antares2 in mouse skin infections, BALB/c mice (*n* = 3 for each dose group) were fully anesthetized with 1% (m/v) pentobarbital sodium (50 mg/kg) and the back hairs were depilated completely with 6% (m/v) sodium sulfide as described^38^. A mixture containing 50 µl of 1 × 10^1^ to 1 × 10^5^ CFU of *S. aureus* USA300/Eno‐Antares2 and 50 µl of HFZ (100 µM) was subcutaneously injected into the dorsal back skin of a mouse. 1 × 10^5^ CFU of wild‐type USA300 injected mice served as negative controls. BL signals were measured using the IVIS® Lumina LT system 1 min after injection of the mixture. The lower limit of sensitivity for skin BL imaging was described as the lowest bacterial CFU in the skin of infected mice that can still emit a bioluminescent signal.

For skin‐tissue BL imaging, BALB/c mice (*n* = 6 per group) were infected subcutaneously with 1 × 10^7^ CFU of *S. aureus* USA300/Eno‐Antares2 in the dorsal skin as described previously^38^. After 6, 12, and 24 h, and 2, 4, and 6 days of infection, each mouse was injected subcutaneously with 100 µl of HFZ (100 µM) around the abscess and imaged. The mice injected with USA300 served as negative controls. After BL imaging, mice were killed, and bacterial burdens in the skin were determined. Total BL flux and skin bacterial CFU were used to assess the correlation between BL signals and skin bacterial CFU loads.

### Determination of HFZ toxicity in mice

Female BALB/c mice (*n* = 3) were subcutaneously infected with 100 µl of HFZ (100 µM) in PBS every day for 11 days. The body weights of the mice were measured every other day. Blood samples were collected 12 h after the last injection of HFZ, and the levels of IL‐6 and TNF‐α in mouse sera were determined using an enzyme‐linked immunosorbent assay (ELISA).

### Evaluation of the antimicrobial efficacy of antibiotics against *S. aureus* infection using BL imaging

According to the linear correlation between in vivo BL signals and bacterial CFU counts, a mouse *S. aureus* skin infection model was used to longitudinally evaluate the antibiotic therapeutic efficacy using USA300/Eno‐Antares2/HFZ BL imaging. Briefly, BALB/c mice (*n* = 5/group) were subcutaneously infected with 1 × 10^7^ CFU of *S. aureus* USA300/Eno‐Antares2 in the dorsal skin. The dose of vancomycin in patients was recommended to be 30 mg/kg/day or greater^49^. Therefore, the mice in the treatment group were each injected intraperitoneally with 8 mg/kg, 16 mg/kg, or 32 mg/kg of ERM or VAN twice a day for 11 days. The NS‐injected mice served as controls. The abscess area was assessed every other day by the maximal length × width of the developing ulcer, as previously described^50^. The body weights of mice were also measured every other day for up to 11 days. On 1, 3, 5, 7, 9, and 11 days postinfection, mice were anesthetized and each mouse was injected subcutaneously with 100 µl of HFZ (100 µM) around the skin abscess. Total BL signals from the dorsal sides of mice were measured using the IVIS® Lumina LT system 3 min after injection of a substrate. Bacterial loads in the skin of mice after 11 days of antibiotic therapy were determined and compared, and IL‐6 levels in mouse sera were determined by enzyme‐linked immunosorbent assay.

### Statistical analysis and reproducibility

Statistical analysis was carried out using GraphPad Prism (GraphPad Software, version 9.2.0.) and SPSS (IBM SPSS Statistics 24). Replicate times and statistical tests for certain experiments are presented in the figure legends. Error bars indicate the standard error of the mean.

## AUTHOR CONTRIBUTIONS

**Weilong Shang**: Data curation (equal); formal analysis (equal); funding acquisition (equal); investigation (equal); methodology (equal); visualization (equal); writing—original draft (lead). **Zhen Hu**: Data curation (equal); methodology (equal); project administration (equal); writing—original draft (supporting). **Mengyang Li**: Data curation (equal); investigation (equal); methodology (equal); visualization (equal); writing—original draft (supporting). **Yuting Wang**: Data curation (equal); methodology (equal); software (equal). **Yifan Rao**: Methodology (equal); software (equal); validation (equal). **Li Tan**: Methodology (supporting); software (supporting); supervision (supporting). **Juan Chen**: Investigation (supporting); methodology (supporting); software (supporting). **Xiaonan Huang**: Investigation (supporting); methodology (supporting); validation (equal). **Lu Liu**: Methodology (supporting); software (supporting). **He Liu**: Data curation (supporting); investigation (supporting); methodology (supporting). **Zuwen Guo**: Investigation (supporting); methodology (supporting); visualization (supporting). **Huagang Peng**: Data curation (supporting); investigation (supporting). **Yi Yang**: Methodology (equal); validation (equal); visualization (equal). **Qiwen Hu**: Methodology (supporting); supervision (supporting). **Shu Li**: Investigation (supporting); supervision (supporting). **Xiaomei Hu**: Conceptualization (equal); supervision (equal); writing—review and editing (supporting). **Jiao Zou**: Conceptualization (supporting); investigation (equal); methodology (equal); supervision (equal); validation (equal); writing—review and editing (supporting). **Xiancai Rao**: Conceptualization (lead); funding acquisition (equal); resources (equal); supervision (equal); writing—review and editing (lead).

## ETHICS STATEMENT

Female BALB/c mice (6–8 weeks old, 16–20 g) were purchased from the Laboratory Animal Center of Third Military Medical University (Army Medical University). All animal experiments were approved by the Institutional Animal Care and Use Committee of the Third Military Medical University (protocol no. #SYXK‐YU‐20170002).

## CONFLICT OF INTERESTS

The authors declare no conflict of interests.

## Supporting information

Supporting information.

## Data Availability

All the data are available in the main text or in the supplementary information materials. All vector information is provided in Table S1.

## References

[1] Chan JD , Bryson‐Cahn C , Kassamali‐Escobar Z , Lynch JB , Schleyer AM . The changing landscape of uncomplicated Gram‐negative bacteremia: a narrative review to guide inpatient management. J Hosp Med. 2020;15:746–753. 32853137 10.12788/jhm.3414

[2] Lagier JC , Dubourg G , Million M , Cadoret F , Bilen M , Fenollar F , et al. Culturing the human microbiota and culturomics. Nat Rev Microbiol. 2018;16:540–550. 29937540 10.1038/s41579-018-0041-0

[3] Culyba MJ , Van Tyne D . Bacterial evolution during human infection: adapt and live or adapt and die. PLoS Pathog. 2021;17:e1009872. 34499699 10.1371/journal.ppat.1009872 PMC8428787

[4] Hou K , Wu ZX , Chen XY , Wang JQ , Zhang D , Xiao C , et al. Microbiota in health and diseases. Signal Transduct Target Ther. 2022;7:135. 35461318 10.1038/s41392-022-00974-4 PMC9034083

[5] Murray C , Ikuta KS , Sharara F , Swetschinski L , Robles Aguilar G , Gray A , et al., Antimicrobial Resistance Collaborators . Global burden of bacterial antimicrobial resistance in 2019: a systematic analysis. Lancet. 2022;399:629–655. 35065702 10.1016/S0140-6736(21)02724-0 PMC8841637

[6] O'Neill J . Tackling drug‐resistant infections globally: final report and recommendations. London: Review on Antimicrobial Resistance; 2016.

[7] Tacconelli E , Carrara E , Savoldi A , Harbarth S , Mendelson M , Monnet DL , et al. Discovery, research, and development of new antibiotics: the WHO priority list of antibiotic‐resistant bacteria and tuberculosis. Lancet Infect Dis. 2018;18:318–327. 29276051 10.1016/S1473-3099(17)30753-3

[8] Marra A . Animal models for drug development for MRSA. Methods Mol Biol. 2020;2069:253–266. 31523778 10.1007/978-1-4939-9849-4_17

[9] Zambito G , Chawda C , Mezzanotte L . Emerging tools for bioluminescence imaging. Curr Opin Chem Biol. 2021;63:86–94. 33770744 10.1016/j.cbpa.2021.02.005

[10] Mezzanotte L , van ‘t Root M , Karatas H , Goun EA , Löwik CWGM . In vivo molecular bioluminescence imaging: new tools and applications. Trends Biotechnol. 2017;35:640–652. 28501458 10.1016/j.tibtech.2017.03.012

[11] Su Y , Walker JR , Park Y , Smith TP , Liu LX , Hall MP , et al. Novel NanoLuc substrates enable bright two‐population bioluminescence imaging in animals. Nat Methods. 2020;17:852–860. 32661427 10.1038/s41592-020-0889-6 PMC10907227

[12] Syed AJ , Anderson JC . Applications of bioluminescence in biotechnology and beyond. Chem Soc Rev. 2021;50:5668–5705. 33735357 10.1039/d0cs01492c

[13] Brodl E , Winkler A , Macheroux P . Molecular mechanisms of bacterial bioluminescence. Comput Struct Biotechnol J. 2018;16:551–564. 30546856 10.1016/j.csbj.2018.11.003 PMC6279958

[14] Li Y , He X , Zhu W , Li H , Wang W . Bacterial bioluminescence assay for bioanalysis and bioimaging. Anal Bioanal Chem. 2022;414:75–83. 34693470 10.1007/s00216-021-03695-9

[15] Kadurugamuwa JL , Modi K , Coquoz O , Rice B , Smith S , Contag PR , et al. Reduction of astrogliosis by early treatment of pneumococcal meningitis measured by simultaneous imaging, in vivo, of the pathogen and host response. Infect Immun. 2005;73:7836–7843. 16299273 10.1128/IAI.73.12.7836-7843.2005 PMC1307043

[16] Pribaz JR , Bernthal NM , Billi F , Cho JS , Ramos RI , Guo Y , et al. Mouse model of chronic post‐arthroplasty infection: noninvasive in vivo bioluminescence imaging to monitor bacterial burden for long‐term study. J Orthop Res. 2012;30:335–340. 21837686 10.1002/jor.21519 PMC3217109

[17] Mesak LR , Qi S , Villanueva I , Miao V , Davies J . *Staphylococcus aureus* promoter‐*lux* reporters for drug discovery. J Antibiot. 2010;63:492–498. 10.1038/ja.2010.74 20606700

[18] Guo Y , Ramos RI , Cho JS , Donegan NP , Cheung AL , Miller LS . In vivo bioluminescence imaging to evaluate systemic and topical antibiotics against community‐acquired methicillin‐resistant *Staphylococcus aureus*‐infected skin wounds in mice. Antimicrob Agents Chemother. 2013;57:855–863. 23208713 10.1128/AAC.01003-12 PMC3553733

[19] Gregor C , Gwosch KC , Sahl SJ , Hell SW . Strongly enhanced bacterial bioluminescence with the *ilux* operon for single‐cell imaging. Proc Natl Acad Sci USA. 2018;115:962–967. 29339494 10.1073/pnas.1715946115 PMC5798359

[20] Yeh HW , Karmach O , Ji A , Carter D , Martins‐Green MM , Ai H . Red‐shifted luciferase‐luciferin pairs for enhanced bioluminescence imaging. Nat Methods. 2017;14:971–974. 28869756 10.1038/nmeth.4400 PMC5678970

[21] Liu S , Su Y , Lin MZ , Ronald JA . Brightening up biology: advances in luciferase systems for in vivo imaging. ACS Chem Biol. 2021;16:2707–2718. 34780699 10.1021/acschembio.1c00549 PMC8689642

[22] Chu J , Oh Y , Sens A , Ataie N , Dana H , Macklin JJ , et al. A bright cyan‐excitable orange fluorescent protein facilitates dual‐emission microscopy and enhances bioluminescence imaging in vivo. Nat Biotechnol. 2016;34:760–767. 27240196 10.1038/nbt.3550 PMC4942401

[23] Miller RJ , Crosby HA , Schilcher K , Wang Y , Ortines RV , Mazhar M , et al. Development of a *Staphylococcus aureus* reporter strain with click beetle red luciferase for enhanced in vivo imaging of experimental bacteremia and mixed infections. Sci Rep. 2019;9:16663. 31723175 10.1038/s41598-019-52982-0 PMC6853927

[24] Hall MP , Unch J , Binkowski BF , Valley MP , Butler BL , Wood MG , et al. Engineered luciferase reporter from a deep sea shrimp utilizing a novel imidazopyrazinone substrate. ACS Chem Biol. 2012;7:1848–1857. 22894855 10.1021/cb3002478 PMC3501149

[25] Suzuki K , Kimura T , Shinoda H , Bai G , Daniels MJ , Arai Y , et al. Five colour variants of bright luminescent protein for real‐time multicolour bioimaging. Nat Commun. 2016;7:13718. 27966527 10.1038/ncomms13718 PMC5171807

[26] Lindberg E , Angerani S , Anzola M , Winssinger N . Luciferase‐induced photoreductive uncaging of small‐molecule effectors. Nat Commun. 2018;9:3539. 30166547 10.1038/s41467-018-05916-9 PMC6117273

[27] Bacconi M , Haag AF , Torre A , Castagnetti A , Chiarot E , Delany I , et al. A stable luciferase reporter plasmid for in vivo imaging in murine models of *Staphylococcus aureus* infections. Appl Microbiol Biotechnol. 2016;100:3197–3206. 26685857 10.1007/s00253-015-7229-2

[28] You Y , Xue T , Cao L , Zhao L , Sun H , Sun B . *Staphylococcus aureus* glucose‐induced biofilm accessory proteins, GbaAB, influence biofilm formation in a PIA‐dependent manner. IJMM. 2014;304:603–612. 24836943 10.1016/j.ijmm.2014.04.003

[29] Bagnoli F , Fontana MR , Soldaini E , Mishra RPN , Fiaschi L , Cartocci E , et al. Vaccine composition formulated with a novel TLR7‐dependent adjuvant induces high and broad protection against *Staphylococcus aureus* . Proc Natl Acad Sci USA. 2015;112:3680–3685. 25775551 10.1073/pnas.1424924112 PMC4378396

[30] Karsi A , Lawrence ML . Broad host range fluorescence and bioluminescence expression vectors for Gram‐negative bacteria. Plasmid. 2007;57:286–295. 17207855 10.1016/j.plasmid.2006.11.002

[31] Shen M , Zhang H , Shen W , Zou Z , Lu S , Li G , et al. *Pseudomonas aeruginosa* MutL promotes large chromosomal deletions through non‐homologous end joining to prevent bacteriophage predation. Nucleic Acids Res. 2018;46:4505–4514. 29514250 10.1093/nar/gky160 PMC5961081

[32] Gaspar N , Walker JR , Zambito G , Marella‐Panth K , Lowik C , Kirkland TA , et al. Evaluation of NanoLuc substrates for bioluminescence imaging of transferred cells in mice. J Photochem Photobiol, B. 2021;216:112128. 33529963 10.1016/j.jphotobiol.2021.112128

[33] Diep BA , Gill SR , Chang RF , Phan TH , Chen JH , Davidson MG , et al. Complete genome sequence of USA300, an epidemic clone of community‐acquired meticillin‐resistant *Staphylococcus aureus* . Lancet. 2006;367:731–739. 16517273 10.1016/S0140-6736(06)68231-7

[34] Rao XC , Li S , Hu JC , Jin XL , Hu XM , Huang JJ , et al. A novel carrier molecule for high‐level expression of peptide antibiotics in *Escherichia coli* . Protein Expr Purif. 2004;36:11–18. 15177279 10.1016/j.pep.2004.01.020

[35] Larsen J , Raisen CL , Ba X , Sadgrove NJ , Padilla‐González GF , Simmonds MSJ , et al. Emergence of methicillin resistance predates the clinical use of antibiotics. Nature. 2022;602:135–141. 34987223 10.1038/s41586-021-04265-w PMC8810379

[36] Wu Y , Wang C , Lin S , Wu M , Han L , Tian C , et al. Octameric structure of *Staphylococcus aureus* enolase in complex with phosphoenolpyruvate. Acta Crystallogr D. 2015;71:2457–2470. 26627653 10.1107/S1399004715018830 PMC4667285

[37] Rack JGM , Morra R , Barkauskaite E , Kraehenbuehl R , Ariza A , Qu Y , et al. Identification of a class of protein ADP‐ribosylating sirtuins in microbial pathogens. Mol Cell. 2015;59:309–320. 26166706 10.1016/j.molcel.2015.06.013 PMC4518038

[38] Shang W , Rao Y , Zheng Y , Yang Y , Hu Q , Hu Z , et al. β‐Lactam antibiotics enhance the pathogenicity of methicillin‐resistant *Staphylococcus aureus* via SarA‐controlled lipoprotein‐like cluster expression. mBio. 2019;10:e00880‐19. 31186320 10.1128/mBio.00880-19 PMC6561022

[39] Yuan J , Yang J , Hu Z , Yang Y , Shang W , Hu Q , et al. Safe staphylococcal platform for the development of multivalent nanoscale vesicles against viral infections. Nano Lett. 2018;18:725–733. 29253342 10.1021/acs.nanolett.7b03893

[40] Mäder U , Nicolas P , Depke M , Pané‐Farré J , Debarbouille M , van der Kooi‐Pol MM , et al. *Staphylococcus aureus* transcriptome architecture: from laboratory to infection‐mimicking conditions. PLoS Genet. 2016;12:e1005962. 27035918 10.1371/journal.pgen.1005962 PMC4818034

[41] Dong X , He Y , Ye F , Zhao Y , Cheng J , Xiao J , et al. Vitamin D3 ameliorates nitrogen mustard‐induced cutaneous inflammation by inactivating the NLRP3 inflammasome through the SIRT3‐SOD2‐mtROS signaling pathway. Clin Transl Med. 2021;11:e312. 33634989 10.1002/ctm2.312 PMC7882108

[42] Jain SK . “Optical Imaging”. Imaging infections: from bench to bedside. Cham, Switzerland: Springer International Publishing; 2017.

[43] Avci P , Karimi M , Sadasivam M , Antunes‐Melo WC , Carrasco E , Hamblin MR . In vivo monitoring of infectious diseases in living animals using bioluminescence imaging. Virulence. 2018;9:28–63. 28960132 10.1080/21505594.2017.1371897 PMC6067836

[44] Rao Y , Peng H , Shang W , Hu Z , Yang Y , Tan L , et al. A vancomycin resistance‐associated WalK(S221P) mutation attenuates the virulence of vancomycin‐intermediate *Staphylococcus aureus* . J Adv Res. 2022;40:167–178. 36100324 10.1016/j.jare.2021.11.015 PMC9481939

[45] Francis KP , Yu J , Bellinger‐Kawahara C , Joh D , Hawkinson MJ , Xiao G , et al. Visualizing pneumococcal infections in the lungs of live mice using bioluminescent *Streptococcus pneumoniae* transformed with a novel Gram‐positive *lux* transposon. Infect Immun. 2001;69:3350–3358. 11292758 10.1128/IAI.69.5.3350-3358.2001 PMC98294

[46] Hofstee MI , Riool M , Terjajevs I , Thompson K , Stoddart MJ , Richards RG , et al. Three‐dimensional *in vitro Staphylococcus aureus* abscess communities display antibiotic tolerance and protection from neutrophil clearance. Infect Immun. 2020;88:e00293‐20. 32817328 10.1128/IAI.00293-20 PMC7573441

[47] Burts ML , Williams WA , DeBord K , Missiakas DM . EsxA and EsxB are secreted by an ESAT‐6‐like system that is required for the pathogenesis of *Staphylococcus aureus* infections. Proc Natl Acad Sci USA. 2005;102:1169–1174. 15657139 10.1073/pnas.0405620102 PMC545836

[48] Shang W , Hu Q , Yuan W , Cheng H , Yang J , Hu Z , et al. Comparative fitness and determinants for the characteristic drug resistance of st239‐MRSA‐III‐t030 and st239‐MRSA‐III‐t037 strains isolated in China. Microb Drug Resist. 2016;22:185–192. 26565599 10.1089/mdr.2015.0226

[49] He N , Su S , Ye Z , Du G , He B , Li D , et al. Evidence‐based guideline for therapeutic drug monitoring of vancomycin: 2020 update by the division of therapeutic drug monitoring, Chinese Pharmacological Society. Clin Infect Dis. 2020;71:S363–S371. 33367582 10.1093/cid/ciaa1536

[50] Wen W , Liu B , Xue L , Zhu Z , Niu L , Sun B . Autoregulation and virulence control by the toxin‐antitoxin system savrs in *Staphylococcus aureus* . Infect Immun. 2018;86:e00032‐18. 29440365 10.1128/IAI.00032-18 PMC5913840

