# Supplementary material for: Optimizing a high‐sensitivity NanoLuc‐based bioluminescence system for in vivo evaluation of antimicrobial treatment
Source: mLife. 2023 Dec 20;2(4):462–78. doi: 10.1002/mlf2.12091 (PMC10989145; doi:10.1002/mlf2.12091)
Supplement: Supplementary file 1 — Supporting information. [file MLF2-2-462-s001.docx]

**Supporting Information for**

**Optimizing a high-sensitivity NanoLuc-based bioluminescence system for *in vivo* evaluation of antimicrobial treatment**

This PDF file includes:

Supplementary Table 1–2.

Supplementary Figure 1–6.

**Supplementary Table 1. Strains and plasmids used in this study**

| **Strain/plasmid** | **Description** | **Reference** |
| --- | --- | --- |
| **Strains** |  |  |
| ***E.coli*** |  |  |
| DH5α | Laboratory strain | Tiangen |
| DH5α/pFH-*nluc* | DH5α carrying the pFH-*nluc* plasmid | This study |
| DH5α/pFH-*lux* | DH5α carrying the pFH-*lux* plasmid | This study |
| ***S. aureus*** |  |  |
| RN4220 | Restriction deﬁcient cloning host | [1] |
| Newman | NCTC 8178, ST8/*agr*-I, highly virulent, extensively used in *S. aureus* animal models | [2] |
| USA300 | ATCC-BAA-1556 (FPR3757), ST8/*agr*-IV, highly virulent, a multidrug-resistant strain extensively used in *S. aureus* animal models | [3] |
| RN4220/pFH-*nluc* | RN4220 carrying the pFH-*nluc* plasmid | This study |
| RN4220/pFH-*lux* | RN4220 carrying the pFH-*lux* plasmid | This study |
| Newman/pFH-*nluc* | Newman carrying the pFH-*nluc* plasmid | This study |
| Newman/pFH-*lux* | Newman carrying the pFH-*lux* plasmid | This study |
| USA300/pFH-*nluc* | USA300 carrying the pFH-*nluc* plasmid | This study |
| USA300/pFH-*lux* | USA300 carrying the pFH-*lux* plasmid | This study |
| USA300/Eno-Nluc | Markerless fused *nluc* with 3′-terminal of *eno* in the genomic DNA of USA300 | This study |
| USA300/Eno-Teluc | Markerless fused *teluc* with 3′-terminal of *eno* in the genomic DNA of USA300 | This study |
| USA300/Eno-Antares2 | Markerless fused *antares2* with 3′-terminal of *eno* in the genomic DNA of USA300 | This study |
| ***P. aeruginosa PAO1*** | | |
| PAO1 | Wild-type *P. aeruginosa* strain PAO1 | [4] |
| PAO1 pUCP24-*nluc* | PAO1 was transformed with the pUCP24-*nluc* plasmid | This study |
|  |  |  |
| **plasmid** |  |  |
| pAKlux2.1 | A *luxCDABE* expression vector | [5] |
| pNL1.1 | NanoLuc genetic reporter vector | Promega |
| pUC-*nluc* | Cloning vector carrying the *nluc* sequence according the codon usage bias of *S. aureus* | This study |
| pUC-*teluc* | Cloning vector carrying the *teluc* sequence according the codon usage bias of *S. aureus* | This study |
| pUC-*antares2* | Cloning vector carrying the *antares2* sequence according the codon usage bias of *S. aureus* | This study |
| PLI50 | *E. coli*–*S. aureus* shuttle overexpression vector, Amp^R^ in *E. coli* and Cm^R^ in *S. aureus* | [6] |
| pFH | pLI50 containing the *S. aureus fhud2* promoter sequence | This study |
| pFH-*nluc* | pFH carrying the *nluc* gene | This study |
| pFH-*lux* | pFH carrying the *luxCDABE* operon | This study |
| pUCP24 | *E. coli*–*P. aeruginosa* shuttle vector, Amp^R^ in *E. coli* and Gm^R^ in *P. aeruginosa* | [4] |
| pUCP24-*nluc* | The *nluc* gene fused with the gentamycin acetyltransferase gene of pUCP24 | This study |
| pBT2 | *E. coli*–*S. aureus* shuttle vector, temperature sensitive, Amp^R^ in *E. coli* and Cm^R^ in *S. aureus* | [7] |
| pBT-eno-*nluc* | pBT2 derivative for *eno*-*nluc* fusion in USA300 | This study |
| pBT-eno-*teluc* | pBT2 derivative for *eno*-*teluc* fusion in USA300 | This study |
| pBT-eno-*antares2* | pBT2 derivative for *eno*-*Antares2* fusion in USA300 | This study |

Amp, ampicillin; Cm, [chloramphenicol](javascript:;); Gm, gentamicin.

**Supplementary Table 2. Primers used in this study**

| **Name** | **Sequence (5′→3′)** | **Product Size (bp)** |
| --- | --- | --- |
| fhud2P-F | cgaggccctttcgtcttcaaattttaaaaggtaaatcgatcgttg | 258 |
| fhud2P-R | gcaggtcgactctagaggatcccataatttcctcctattgaaaatg |  |
| nluc-F | caataggaggaaattatggttttcacgttagaagatttcg | 555 |
| nluc-R | atgcctaaaaacctacagctcgagttaagctaaaatacg |  |
| teluc-F | ttcaataggaggaaattatggttttcacgttagaagatttc | 555 |
| teluc-R | gcctaaaaacctacagctcgagttaagctaaaatacgttcgtgtaaacg |  |
| lux-F | ttcaataggaggaaattatggcaaatatgactaaaaaaatttc | 5848 |
| lux-R | gttttatgcctaaaaacctacagtcaactatcaaacgcttcg |  |
| nluc-F1 | taccgccaccatggttttcacgttagaagatttc | 533 |
| nluc-R1 | cgaattgttaagctaaaatacgttcacataaacg |  |
| Up-eno-F1 | tcgagctcggtacccgggatgatcgcattagacggta | 1045 |
| Up-eno-R1 | tgaaaaccattttatctaagttatagaatgatttg |  |
| Down-eno-F1 | ctcgagttttctttataatcaaatgctgac | 970 |
| Down-eno-R1 | gataaactaccgcattactgcttttaccttcttggag |  |
| eno-nluc-F1 | cttagataaaatggttttcacgttagaagatttc | 546 |
| eno-nluc-R1 | gattataaagaaaactcgagttaagctaaaatacg |  |
| Up-eno-F2 | cgagctcggtacccgggatgatcgcattagacggtac | 1043 |
| Up-eno-R2 | gaaaaccattttatctaagttatagaatgatttgataccgtc |  |
| Down-eno-F2 | cttaactcgagttttctttataatcaaatgctgac | 973 |
| Down-eno-R2 | taaactaccgcattactgcttttaccttcttggagtag |  |
| eno-teluc-F2 | gataaaatggttttcacgttagaagatttcg | 544 |
| eno-teluc-R2 | ttgattataaagaaaactcgagttaagctaaaatacgttcgtgtaaacg |  |
| Up-eno-F3 | gagctcggtacccgggatgatcgcattagacggt | 1044 |
| Up-eno-R3 | tgctcaccattttatctaagttatagaatgatttgataccg |  |
| Down-eno-F3 | caagtgactcgagttttctttataatcaaatgctgac | 977 |
| Down-eno-R3 | gataaactaccgcattactgcttttaccttcttggagtag |  |
| eno-antares2-F3 | cttagataaaatggtgagcaagggcgag | 1905 |
| eno-antares2-R3 | gaaaactcgagtcacttgtacagctcgtccatgc |  |
| pBT2-PCR-F | ccgggtaccgagctcg | 7193 |
| pBT2-PCR-R | taatgcggtagtttatcacagtt |  |
| pLI50-PCR-F | ttgaagacgaaagggcctcg | 5484 |
| pLI50-PCR-R | ggatcctctagagtcgacctgc |  |
| pUC24-PCR-F | tattttagcttaacaattcgttcaagccgag | 4056 |
| pUC24-PCR-R | tgaaaaccatggtggcggtacttgggtc |  |

**
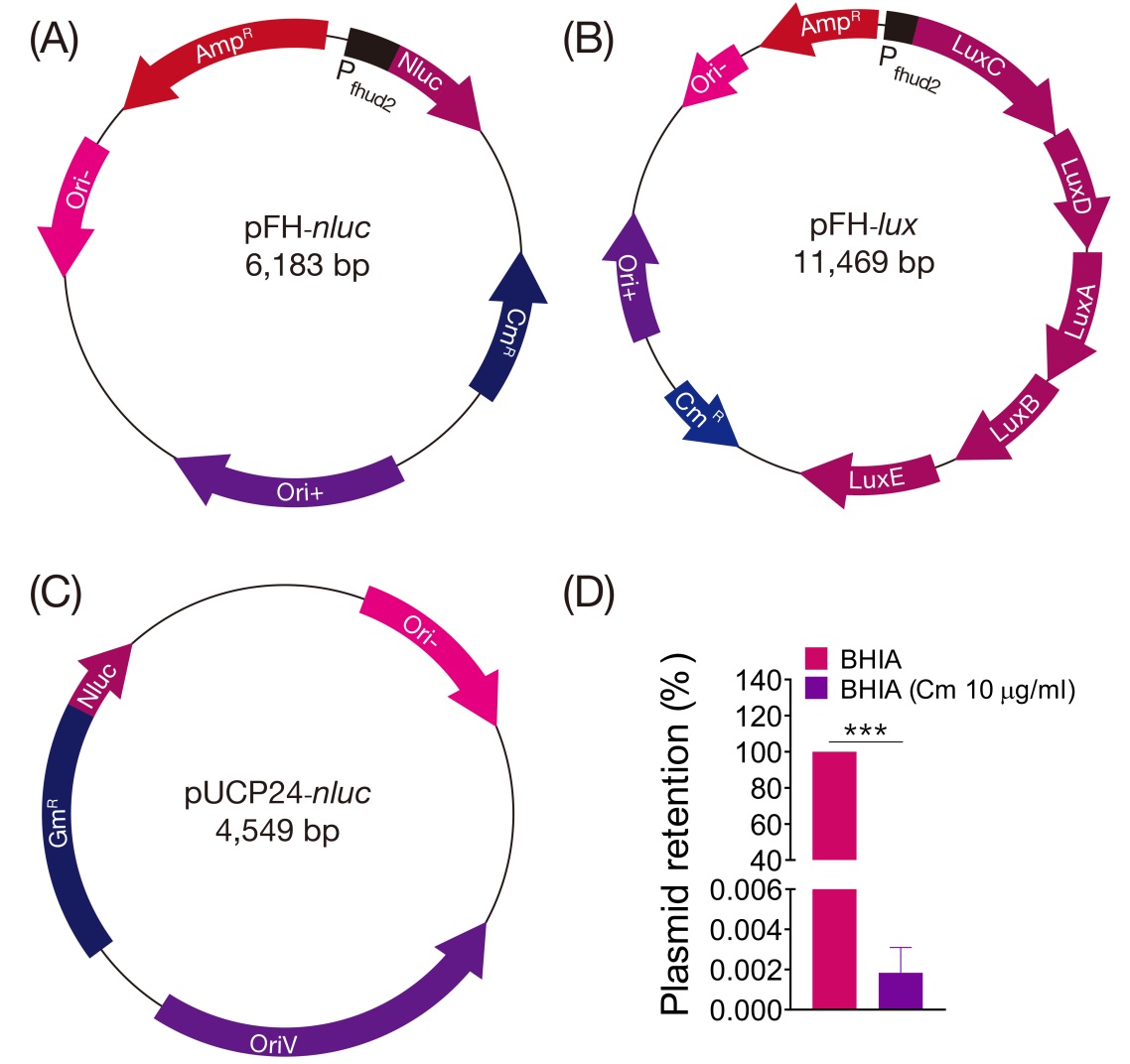
**

**Supplementary** **Figure 1. Schematic maps of plasmid constructs and the *in vivo* stability of pFH-*nluc* plasmid in *S. aureus*.** Schematic plasmid maps of **(A)** pFH-*nluc* and **(B)** pFH-*lux* under the control of the *fhuD2* gene promoter (P_fhuD2_), and **(C)** pUCP24-*nluc*. Replication origins for *S. aureus* (Ori+), *E. coli* (Ori-), and *P. aeruginosa* (OriV), as well as Amp^R^, Gm^R^, and Cm^R^ antibiotic resistance genes for the selection of recombinant bacteria are indicated. **(D)** Evaluation of *in vivo* plasmid retention in *S. aureus* strain USA300 transformed with pFH-*nluc*. Bacteria were recovered from the kidneys of mice (*n* = 6) 5 days after intravenous injection. The bacterial loads were counted by plate dilution method with BHIA and BHIA supplemented with 10 µg/mL of chloramphenicol (Cm). The mean number of viable bacteria grown on BHIA plates was adjusted to 100%, while the relative number of viable bacteria grown on BHIA plates carrying Cm was calculated and indicated. Statistical significance was analyzed by Unpaired two-tailed *t*-test, ****P* < 0.001. Data were presented as mean ± standard error of the mean (SEM).

**
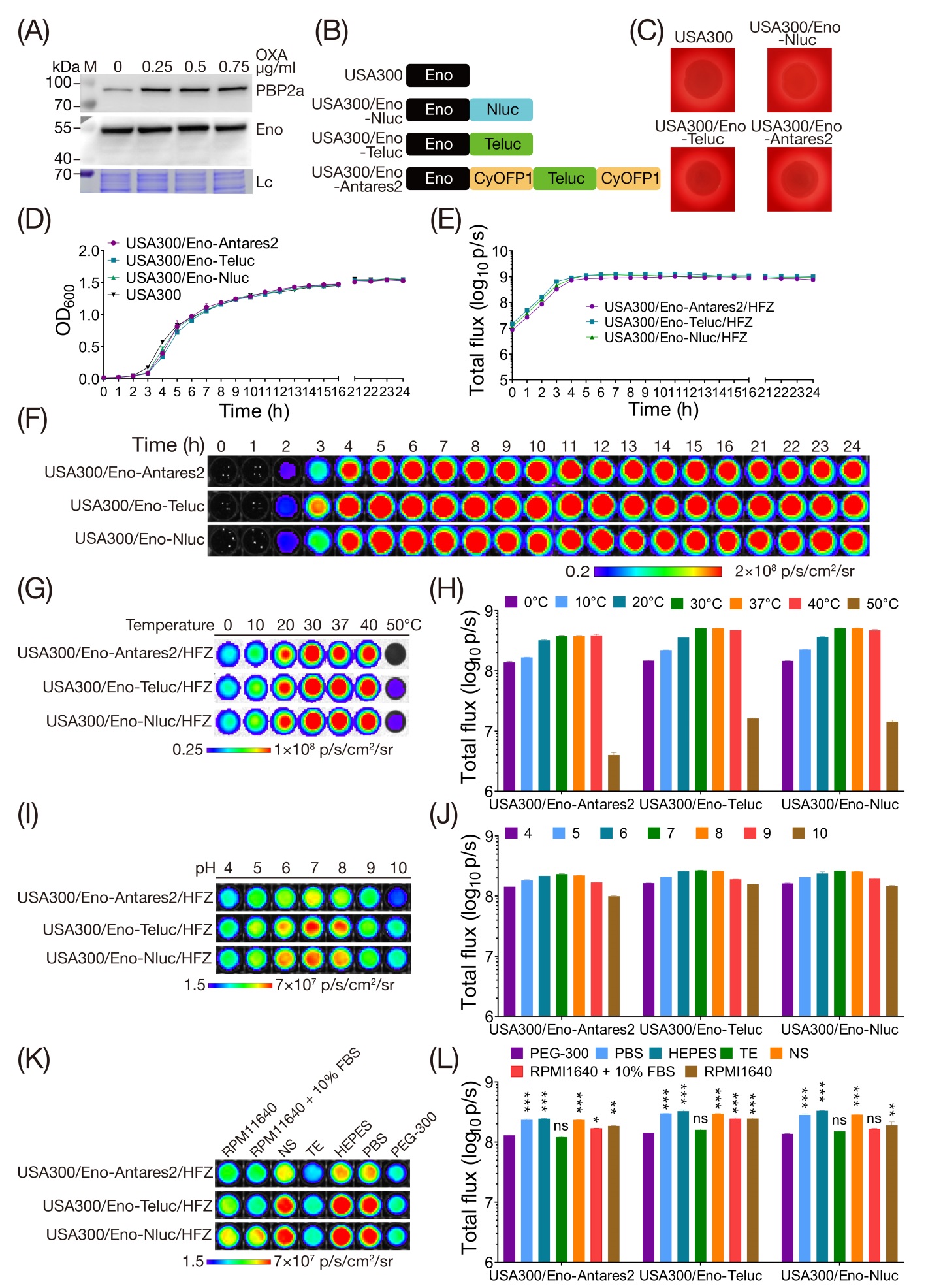
**

**Supplementary** **Figure 2. Characterization of Nluc-based luciferase expressing *S. aureus* reporter strains *in vitro*. (A)** Western blot analysis of the expression of PBP2a and Eno in *S. aureus* USA300 treated with different concentrations of OXA as indicated. The protein gel served as loading control (Lc), and the molecular weights of the protein markers (M) were indicated on the left. **(B)** Schematic diagram of *S. aureus* USA300 and Eno fused Nluc-based luciferases in the reporter strains. **(C)** Hemolytic experiments. **(D)** Growth curves of *S. aureus* reporter strains. Data were presented as mean ± SEM. **(E)** BL intensity for the reporter strains over time. Data were presented as mean ± SEM. **(F)** Representative BL images of 50 µL of 1:100 diluted bacterial culture mixed with 50 µL of HFZ (100 µM) over time. **(G)** BL images of 50 µL of *S. aureus* reporter strains (1 × 10^6^ CFU/mL) mixed with 50 µL of HFZ (100 µM) in a thermostatic metal bath under different temperatures (0–50 °C). **(H)** Quantification of BL intensity. Data were presented as mean ± SEM. **(I)** *S. aureus* reporter strains were adjusted to 1 × 10^6^ CFU/mL with PBS at different pH (4–10), and BL images of 50 µL of bacterial suspension mixed with 50 µL of HFZ (100 µM). **(J)** Quantification of BL intensity. Data were presented as mean ± SEM. **(K)** BL images of 50 µL of *S. aureus* reporter strains (1 × 10^6^ CFU/mL) mixed with 50 µL of HFZ (100 µM) in different physiochemical conditions, including PEG-300 formulation, [HEPES](https://www.beyotime.com/product/C0215-100ml.htm), TE, normal saline, RPMI 1640, or RPMI 1640 plus 10% FBS. **(L)** Quantification of BL intensity. Data were presented as mean ± SEM. Statistical significance was analyzed by Two-way ANOVA, ns indicates no significance, **P* < 0.05, ***P* < 0.01, and ****P* < 0.001.

**
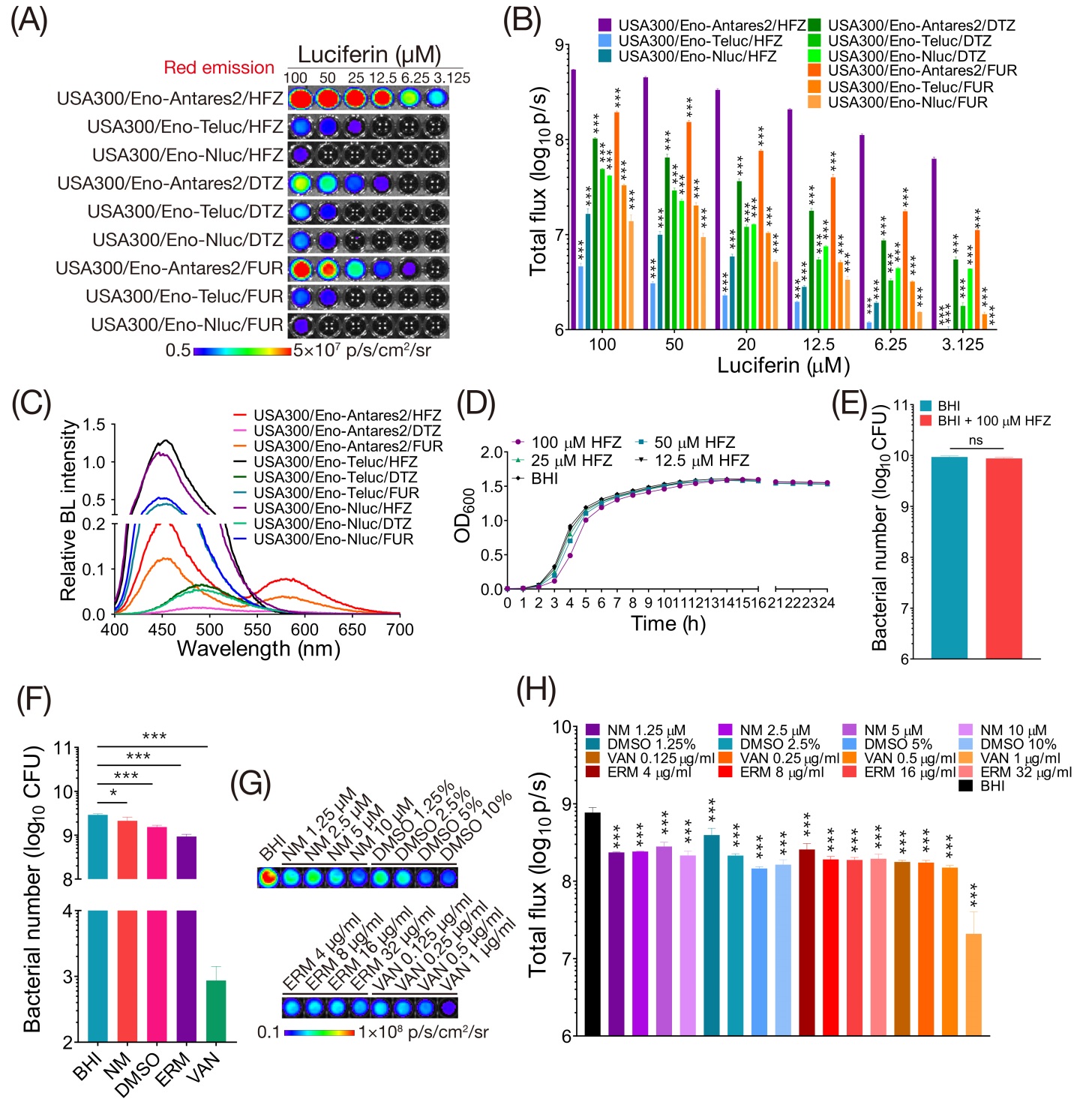
**

**Supplementary** **Figure 3. Performance of *S. aureus* reporter strains *in vitro* BL imaging.**

**(A)** 50 µL of bacterial culture (1 × 10^7^ CFU/mL) in the presence of various substrates as indicated. Images were acquired with an emission filter of 591 ± 10 nm. **(B)** Quantification of BL intensity. Data were presented as mean ± SEM. Statistical significance was analyzed by Two-way ANOVA, ****P* < 0.001. **(C)** BL emission spectra of 100 µL of bacterial culture (1 × 10^9^ CFU/mL) reacted with 10 µL of various substrates (3 mM). The spectra were normalized to the Nluc emission peak at 460 nm. **(D)** Growth curves of the USA300/Eno-Antares2 strain cultured in fresh BHI broth containing a range of concentrations (12.5, 25, 50, and 100 µM) of HFZ. Data were presented as mean ± SEM. **(E)** Bacterial CFU was counted on BHI agar plates. Statistical significance was analyzed by Unpaired two-tailed *t*-test, ns indicates no significance. **(F)** Bacterial counts of USA300/Eno-Antares2 cultured in fresh BHI broth containing 10 µM of nitrogen mustard (NM), 10% of dimethyl sulfoxide (DMSO), 32 μg/mL of erythromycin (ERM), and 1 μg/mL of vancomycin (VAN). Data were presented as mean ± SEM. Statistical significance was analyzed by One-way ANOVA, **P* < 0.05, ****P* < 0.001. **(G)** BL images of 50 µL of a 1:100 dilution of USA300/Eno-Antares2 culture mixed with 50 µL of HFZ (100 µM) in a black 96-well plate. **(H)** Quantification of BL intensity. Data were presented as mean ± SEM. Statistical significance was analyzed by One-way ANOVA, ****P* < 0.001.


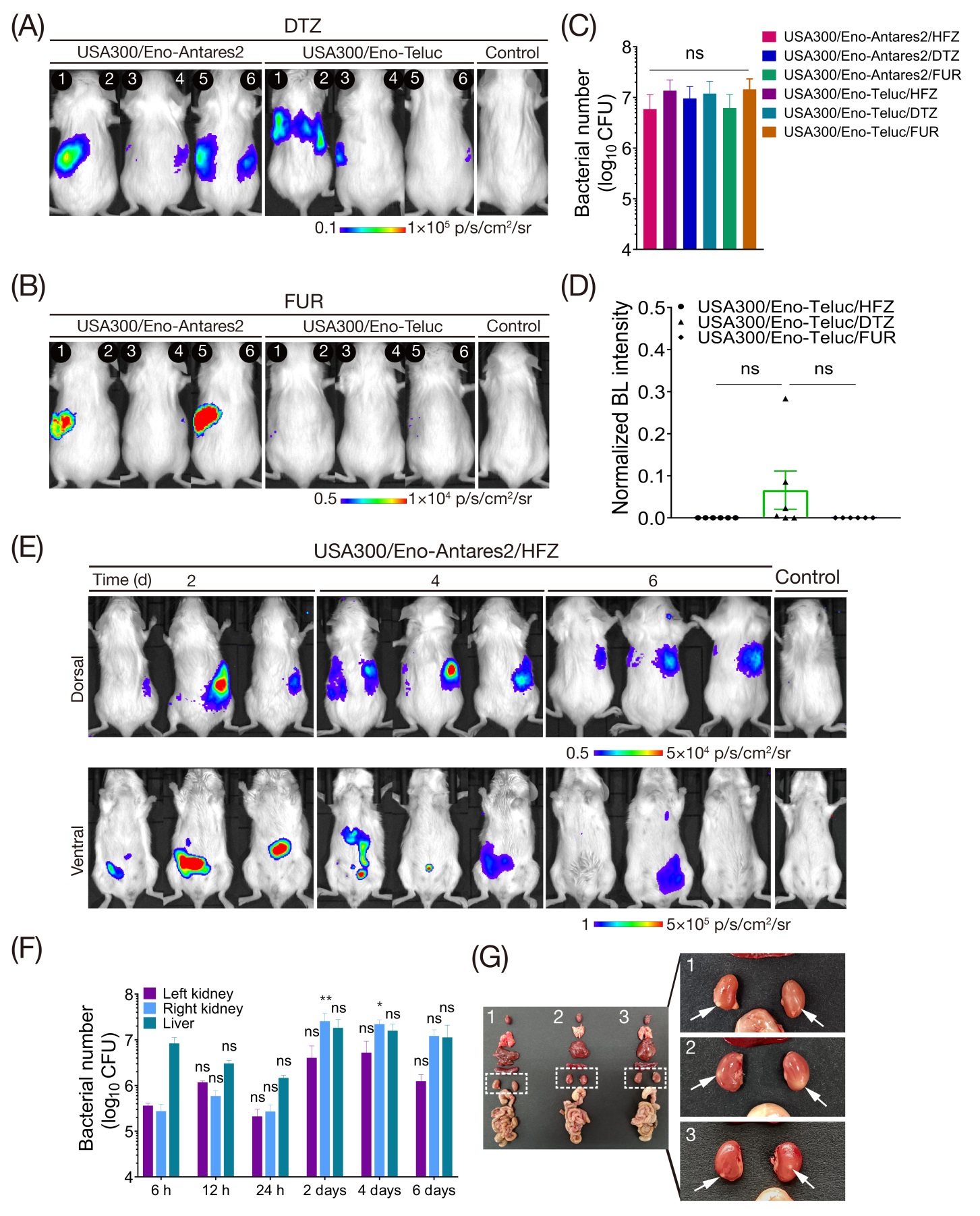


**Supplementary** **Figure 4.** **Deep-tissue BL imaging with USA300/Eno-Antares2 and USA300/Eno-Teluc reporters.** BL imaging in BALB/c mice (*n* = 3) infected intravenously with 1 × 10^7^ CFU of USA300/Eno-Antares2 or USA300/Eno-Teluc. After 24 h of infection, 1 µmol of **(A)** DTZ or **(B)** FUR was injected intraperitoneally, and BLI signals were measured. The numbers 1–6 represent the number of left and right kidneys in mice. **(C)** Bacterial loads in the kidneys of infected mice after 24 h of infection. Data were presented as mean ± SEM. Statistical significance was analyzed by One-way ANOVA, ns indicates no significance. **(D)** Quantitative comparison of the normalized BL intensity of USA300/Eno-Teluc with different substrates. Data were presented as mean ± SEM. Statistical significance was analyzed using two-tailed Mann Whitney U test, ns indicates no significance. **(E)** BL images of mice on 2, 4, and 6 days after being intravenously injected with 1 × 10^7^ CFU of USA300/Eno-Antares2. 1 µmol of HFZ was injected intraperitoneally, and BLI signals were captured. **(F)** Bacterial CFU counts from left/right kidneys and livers of mice at 6, 12, and 24 h, and 2, 4, and 6 days after infection of 1 × 10^7^ CFU of USA300/Eno-Antares2. Data were presented as mean ± SEM. Statistical significance was analyzed by Two-way ANOVA, ns indicates no significance, **P* < 0.05, and ***P* < 0.01. **(G)** Gross specimens of the heart, lung, liver, spleen, kidney, and intestine from infected mice 6 days after infection with 1 × 10^7^ CFU of USA300/Eno-Antares2. The white arrows indicate the formation of abscesses in the kidneys*.*


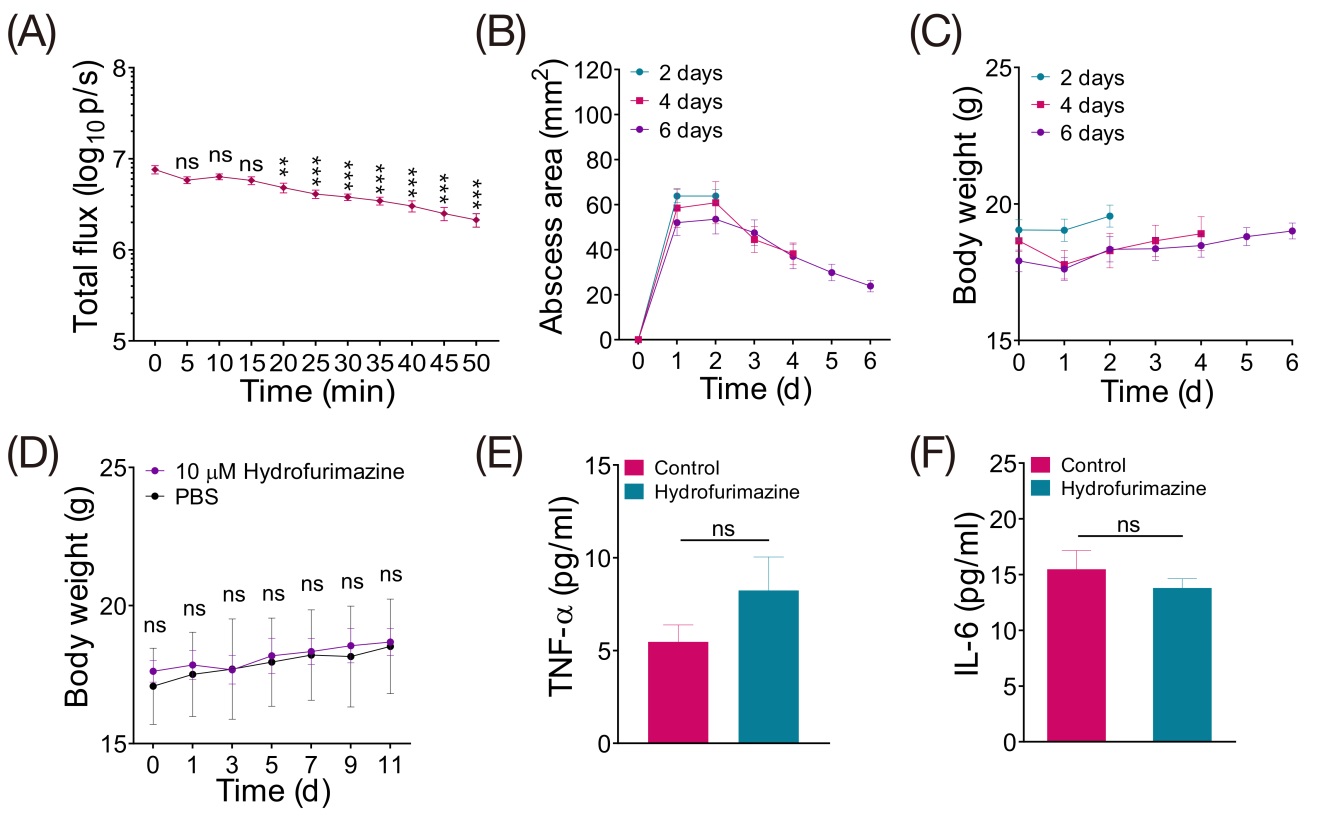


**Supplementary** **Figure 5. BL signals of USA300/Eno-Antares2/HFZ combination in the mouse skin infection model.** **(A)** BL signal stability of mice injected with *S. aureus* USA300/Eno-Antares2 and substrate HFZ. Mice were subcutaneously injected with a mixture of 50 µL of USA300/Eno-Antares2 (1 × 10^3^ CFU) and 50 µL of HFZ (100 µM) (*n* = 3/group), and BL signals were monitored for every 5 min up to 50 min. Data were presented as mean ± SEM. Statistical significance was analyzed by One-way ANOVA, ns indicates no significance, ***P* < 0.01, and ****P* < 0.001. Mice (*n* = 6 for each group of 2, 4, or 6 days) were subcutaneously infected with 1 × 10^7^ CFU of reporter strain USA300/Eno-Antares2, and **(B)** the skin abscess areas and **(C)** body weights of the infected mice were measured every day. Data were presented as mean ± SEM. BALB/c mice (*n* = 5/group) received a subcutaneous infection of 1 × 10^7^ CFU of USA300/Eno-Antares2. **(D)** BALB/c (*n* = 3) mice were injected subcutaneously with 100 µL of HFZ (100 µM) every day for 11 days. The body weights of mice were measured every other day. Data were presented as mean ± SEM. Statistical significance was analyzed by Multiple *t* test, ns indicates no significance. **(E)** TNF-α and **(F)** IL-6 levels in mouse sera were determined by ELISA. Mice administered with PBS served as the negative controls. Data were presented as mean ± SEM. Statistical significance was analyzed by Unpaired Two-tailed *t*-test, ns indicates no significance.

**
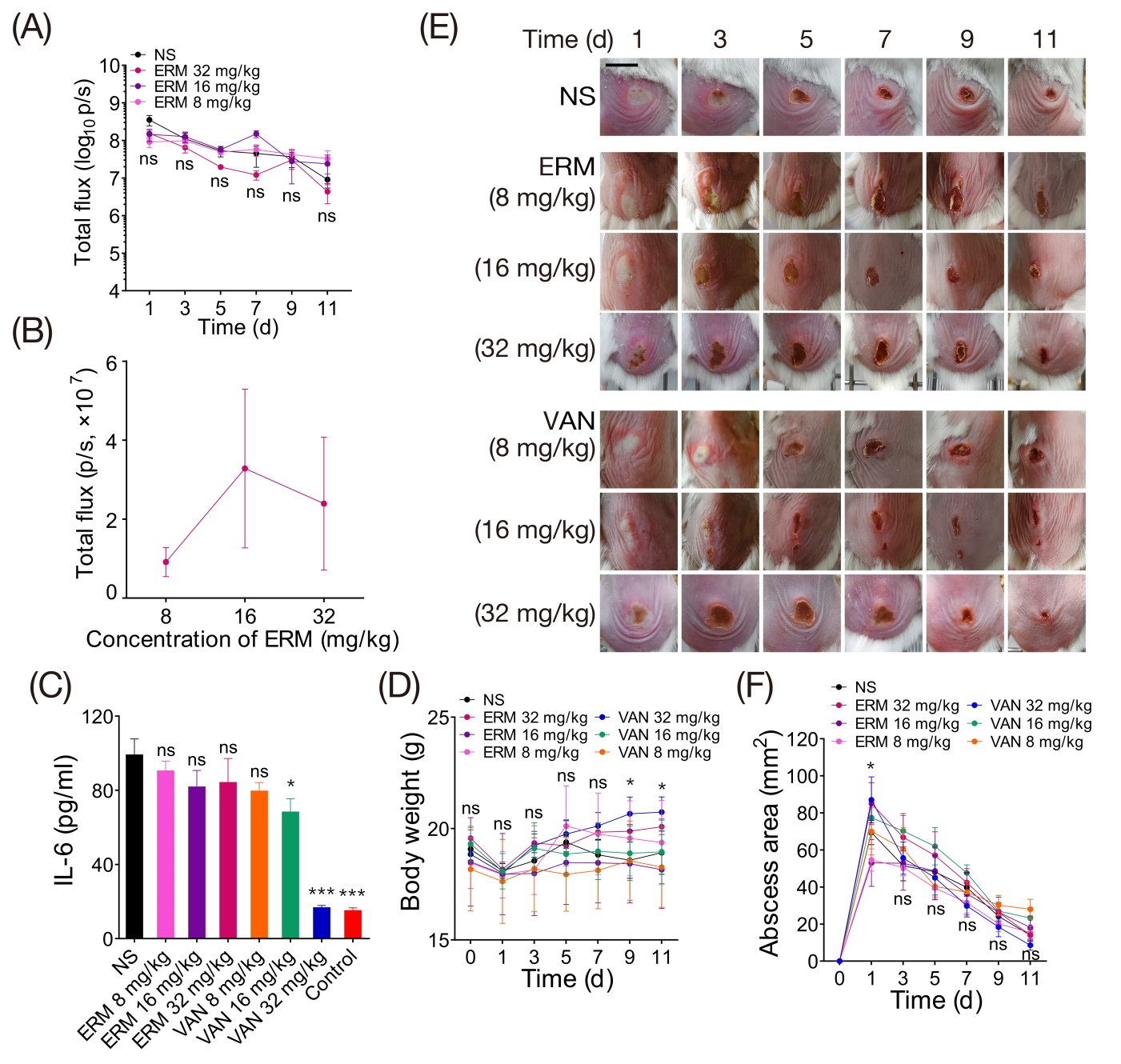
**

**Supplementary** **Figure 6.** **Evaluation of antimicrobial efficacy of antibiotics against *S. aureus* skin infection by using *in vivo* BL imaging.** **(A)** BL intensities from each mouse in groups of normal saline-treated and different concentrations of ERM-treated on indicated time were measured and presented. Data were presented as mean ± SEM. Statistical significance was analyzed by two-tailed Mann Whitney U test, ns indicates no significance relative to the normal saline-treated group at the same time. **(B)** Correlation of BL intensities in the skin abscesses with the concentrations of VAN in groups of ERM-treated at 11 days postinfection of 1 × 10^7^ CFU of USA300/Eno-Antares2. Data were presented as mean ± SEM. **(C)** IL-6 levels in mouse sera were determined after 11 days of antibiotic therapy. Uninfected mice administered with PBS served as the negative controls. Data were presented as mean ± SEM. Statistical significance was analyzed by One-way ANOVA, ns indicates no significance, **P* < 0.05, ****P* < 0.001. **(D)** Mouse weights were determined every other day during treatment. Data were presented as mean ± SEM. Statistical significance was analyzed by Two-way ANOVA, ns indicates no significance, **P* < 0.05. **(E)** Representative skin abscesses in mice after treatment with normal saline, different concentrations of ERM or VAN at the time indicated. Scale bar = 1 cm. **(F)** The skin abscess areas of the treated mice were measured every other day. Data were presented as mean ± SEM. Statistical significance was analyzed by Two-way ANOVA, ns indicates no significance, **P* < 0.05.

**References**

**1.** Berscheid A, Sass P, Weber-Lassalle K, Cheung AL, Bierbaum G. Revisiting the genomes of the *Staphylococcus aureus* strains NCTC 8325 and RN4220. Int J Med Microbiol. 2012; 302: 84–87.

**2.** Duthie ES, Lorenz LL. Staphylococcal coagulase; mode of action and antigenicity. J Gen Microbiol. 1952; 6: 95–107.

**3.** Diep BA, Gill SR, Chang RF, Phan TH, Chen JH, Davidson MG, et al. Complete genome sequence of USA300, an epidemic clone of community-acquired meticillin-resistant *Staphylococcus aureus*. Lancet. 2006; 367: 731–739.

**4.** Shen M, Zhang H, Shen W, Zou Z, Lu S, Li G, et al. *Pseudomonas aeruginosa* MutL promotes large chromosomal deletions through non-homologous end joining to prevent bacteriophage predation. Nucleic Acids Res. 2018; 46: 4505–4514.

**5.** Karsi A, Lawrence ML. Broad host range fluorescence and bioluminescence expression vectors for Gram-negative bacteria. Plasmid. 2007; 57: 286–295.

**6.** You Y, Xue T, Cao L, Zhao L, Sun H, Sun B. *Staphylococcus aureus* glucose-induced biofilm accessory proteins, GbaAB, influence biofilm formation in a PIA-dependent manner. Int J Med Microbiol. 2014; 304: 603–612.

**7.** Shang W, Rao Y, Zheng Y, Yang Y, Hu Q, Hu Z, et al. β-Lactam antibiotics enhance the pathogenicity of methicillin-resistant *Staphylococcus aureus* via SarA-controlled lipoprotein-like cluster expression. mBio. 2019; 10: e00880-19.
